# A multi-tissue full lifespan epigenetic clock for mice

**DOI:** 10.18632/aging.101590

**Published:** 2018-10-21

**Authors:** Michael J. Thompson, Karolina Chwiałkowska, Liudmilla Rubbi, Aldons J. Lusis, Richard C. Davis, Anuj Srivastava, Ron Korstanje, Gary A. Churchill, Steve Horvath, Matteo Pellegrini

**Affiliations:** 1 Molecular, Cell and Developmental Biology, University of California Los Angeles, Los Angeles, CA 90095, USA; 2 Centre for Bioinformatics and Data Analysis, Medical University of Bialystok, Bialystok, Poland; 3 Department of Microbiology, Immunology and Molecular Genetics, Department of Medicine, and Department of Human Genetics, University of California Los Angeles, Los AngelesCA 90095, USA; 4 The Jackson Laboratory, Farmington, CT 06032, USA; 5 The Jackson Laboratory, Bar Harbor, Maine 04609, USA; 6 Department of Human Genetics and Biostatistics, University of California Los Angeles, Los AngelesCA 90095, USA; * Equal contribution

**Keywords:** epigenetic clock, biological age, mouse, DNA methylation

## Abstract

Human DNA-methylation data have been used to develop highly accurate biomarkers of aging ("epigenetic clocks"). Recent studies demonstrate that similar epigenetic clocks for mice (*Mus Musculus*) can be slowed by gold standard anti-aging interventions such as calorie restriction and growth hormone receptor knock-outs. Using DNA methylation data from previous publications with data collected in house for a total 1189 samples spanning 193,651 CpG sites, we developed 4 novel epigenetic clocks by choosing different regression models (elastic net- versus ridge regression) and by considering different sets of CpGs (all CpGs vs highly conserved CpGs). We demonstrate that accurate age estimators can be built on the basis of highly conserved CpGs. However, the most accurate clock results from applying elastic net regression to all CpGs. While the anti-aging effect of calorie restriction could be detected with all types of epigenetic clocks, only ridge regression based clocks replicated the finding of slow epigenetic aging effects in dwarf mice. Overall, this study demonstrates that there are trade-offs when it comes to epigenetic clocks in mice. Highly accurate clocks might not be optimal for detecting the beneficial effects of anti-aging interventions.

## Introduction

Our understanding of age-related epigenetic changes in DNA-methylation in humans has progressed rapidly with the technical advancement of genomic platforms [1–14]. For mammalian genomes, DNA methylation is a modification that regulates gene expression via its presence or absence at gene promoters and enhancers. During development, germline DNA methylation is erased, but re-established in tissue-specific patterns as tissue development programs unfold after implantation [15]. Age-based methylation changes accompany the functional decline of adult stem cells [16–18], and even small changes can lead to loss of regulatory control of gene transcription, either directly or via additive effects [19].

The correlation between chronological age and DNA methylation over the course of an entire lifespan is strong [20–23]. Recent studies have taken advantage of this relationship to accurately estimate chronological age based on the methylation levels of multiple CpG dinucleotides [10,13,24]. For example, the human multi-tissue epigenetic age estimation method combines the weighted average of DNA methylation levels of 353 CpGs into an age estimate that is referred to as DNAm age or epigenetic age [13]. Most importantly, we and others have shown that human epigenetic age relates to biological age, not just chronological age. This is demonstrated by the finding that the discrepancy between DNAm age and chronological age (what we term “epigenetic age acceleration”) is predictive of all-cause mortality even after adjusting for a variety of known risk factors [25–29]. Epigenetic age acceleration is associated with lung cancer risk [30], cognitive and physical functioning [31], Alzheimer's disease [32], centenarian status [29,33], Down syndrome [34], Werner Syndrome [35], HIV infection [36], Huntington's disease [37], obesity [38], menopause [39], osteoarthritis [40], and Parkinson's disease [41]. Moreover, we have demonstrated that the human epigenetic clock applies without change to chimpanzees [13] but it loses utility for other animals as a results of evolutionary genome sequence divergence. Moving beyond primates into the broader mammalian arena, we recently constructed an epigenetic clock for canids using DNA-methylation data from *Canis familiaris* (domesticated dog) and *Canis lupus* (wolf) [42].

Recently, other groups constructed epigenetic clocks for mice and used these to evaluate gold standard longevity interventions [43]. Petkovich, et al., derived a clock from blood samples of approximately 250 mice in order to examine changes induced by diet treatments and changes associated with genetic backgrounds that produce dwarfism (and long-lived) phenotypes [44]. Similarly, Cole, et al. examined the effects of genetic background (dwarf genotypes) and diet interventions on longevity, and their data was utilized by Wang, et al. to construct a DNA-methylation clock [45,46]. Stubbs, et al, developed a clock for multiple tissue types. Application of their clock to samples from experimental interventions yielded biologically meaningful differences in epigenetic age [47]. Overall, these independent publications led to the important insight that epigenetic clocks for mice detect anti-epigenetic aging effects of gold standard interventions such as calorie restriction and growth hormone receptor knockouts.

Our current study addressed the following aims. First, to develop a multi-tissue DNA-methylation based estimator of chronological age across the entire lifespan based on new and existing reduced representation bisulfite sequencing (RRBS) data. Second, to evaluate the robustness of reported findings surrounding gold standard anti-aging interventions using the novel epigenetic clocks. Third, to assess whether one can develop an epigenetic clock based on roughly 1k CpGs in evolutionarily conserved genomic regions.

To address these aims, we combined hundreds of new DNA-methylation samples collected from several mouse tissues with publicly available data from previous studies of mouse DNA-methylation. These data include samples obtained with RRBS and whole-genome bisulfite sequencing (WGBS). We compared clocks built with different regression methods using hundreds of thousands of CpGs as input as well as a clock constructed from a limited set of mammalian-conserved CpGs. We evaluated the performance of these clocks across samples and tissues. We applied the most accurate clock to samples from previous longevity studies of mice to measure the effects of these interventions on epigenetic aging. And, finally, we performed a GWAS analysis using epigenetic age as a trait in a subset of age-matched mouse samples covering 88 strains.

## RESULTS

### Data set

Based on calculations and criteria described in the Methods section, we constructed a matrix of high confidence methylation levels for 1189 mouse samples at 193,651 CpG sites. Of these 1189 mice, 893 were used as the training set for regression models described below. This was the largest matrix we could construct while minimizing missing values to 2% of total. The remaining 296 samples were held out entirely from the training so they could be used to investigate the effects of the experimental treatments (e.g. calorie restriction) and growth hormone receptor knockouts.

### Four different epigenetic clocks

We considered 4 types of epigenetic clocks. The first two clocks are constructed on the basis of all 193,651 CpG sites (covariates). In particular, the "elastic net clock" used an elastic net model to regress chronological age (dependent variable) on all methylation levels. The second clock ("ridge regression clock") used a ridge regression model instead of an elastic net regression model. The two "conserved" clocks were constructed using elastic net regression and ridge regression, respectively, using 952 highly conserved CpGs, i.e. located in highly conserved stretches of DNA (Methods).

### Accuracy with respect to chronological age

We compared the four different epigenetic clocks with respect to estimating chronological age at the time of DNA sample collection (Table 1). The training set estimates of accuracy are overly optimistic and should be ignored. To arrive at unbiased estimates of the age correlation R (defined as Pearson correlation between DNAm age and chronological age) and the median absolute error (mae), the table reports three types of cross-validation estimates: i) leave-one-batch-out estimate (row "batch" in Table 1), ii) leave-one-sample-out estimate (row "sample" in Table 1), and iii) a 10 fold cross validation estimate. The three different cross validation estimates lead to the same conclusion: elastic net regression outperforms the other clocks when it comes to CV estimates of age correlations and median error. For example, the elastic net clock leads to a (leave-one-batch-out) age correlation of R=0.82 and a median error of 2.5 months. Although the conserved clocks are clearly inferior to those based on all CpGs, their accuracy remains impressive. For example, the elastic net conserved clock leads to a (leave-one-batch-out) age correlation of R=0.68 and a median error of 3.8 months.

**Table 1:** Summary performance statistics of epigenetic aging models (“clocks”).

**CpGs**	**Estimate**	**Regression**	**Age cor.**	**mae**	**mean model size**	**model size std dev**
All CpGs Clock	Training set	Ridge	1.00	0.1	193651	0
		Elastic net	0.99	0.7	582	0
	LO-Batch-Out	Ridge	0.79	3.1	193641	0
		Elastic	0.82	2.5	529	81
	LO-Sample-Out	Ridge	0.85	2.1	193651	0
		Elastic	0.89	1.8	444	81
	10-fold CV	Ridge	0.88	0.3	193641	0
		Elastic	0.89	1.2	463	134
Conserved CpGs Clock	Training	Ridge	0.85	2.7	952	0
		Elastic	0.91	1.9	274	0
	LO-Batch-Out	Ridge	0.64	4.0	952	0
		Elastic	0.68	3.8	214	39
	LO-Sample-Out	Ridge	0.75	3.3	952	0
		Elastic	0.78	2.4	236	6
	10-fold CV	Ridge	0.77	3.5	952	0
		Elastic	0.80	2.5	247	23

Accuracy of estimating chronological age for 4 different epigenetic clocks. The 4 clocks differ in terms of the CpGs that were used in their construction (first column) and in terms of the underlying regression model (third column). The second column describes the method for estimating the predictive accuracy. The training set estimates are overly optimistic and should be ignored. Leave-one-batch out estimates and leave-one-sample-out estimates provide accuracy estimates that are far less biased than those obtained in the training set. The mean model size refers to the number of CpGs selected by the penalized regression model. Since the ridge regression is based on all CpGs, the standard deviation is zero.

**Figure 1:**
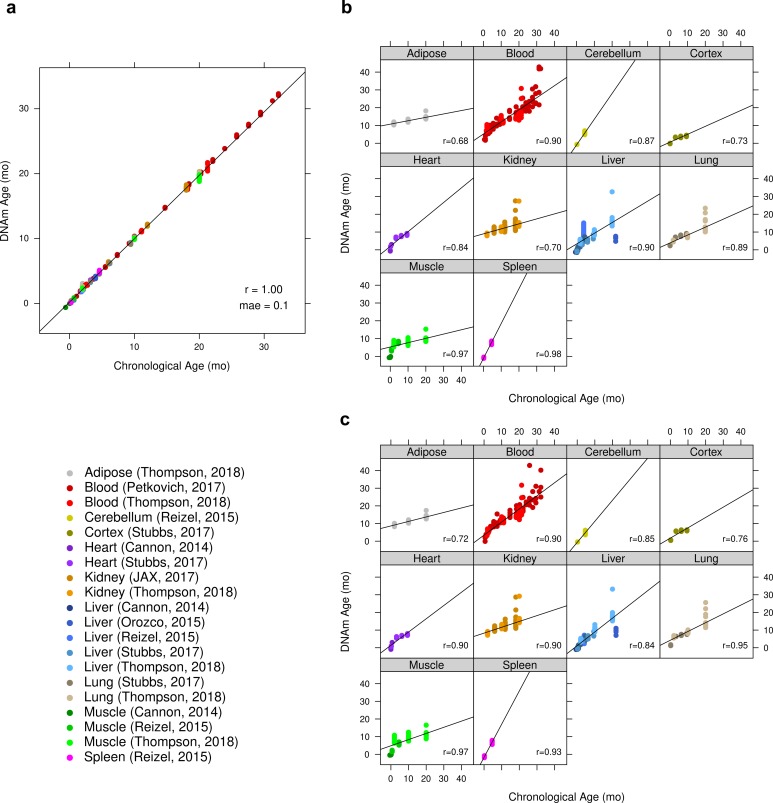
**Accuracy of ridge regression epigenetic age predictions.** DNA methylation age (y-axis) versus chronological age (x-axis) for all mouse samples. (**a**) Performance of ridge regression clock based on all 192K CpGs in all training samples. The training set estimates of the accuracy are overly optimistic and should be ignored. (**b**) Results by tissue type of cross-validated predictions obtained by iteratively withholding one “batch” (tissue x publication). For the batch cross-validation of this clock, the global Pearson correlation between predicted and chronological age was 0.79 (p < 2E-195) with a mae of 3.1 months. All models in these iterative cross-validations had the same size of 193,651 CpGs. (**c**) Scatter plots by tissue type based on DNAm age estimates made with an iterative leave-one-sample-out cross-validation. The correlation between predicted and chronologic age was 0.85 (p < 6E-258) with a mae of 2.1 months.

We find that these epigenetic clocks are multi-tissue clocks, i.e. they lead to accurate age estimates in all considered tissues: results for the ridge regression clock based on all CpGs can be found Fig. 1. Analogous results for elastic net clocks based on all CpGs or based on only conserved CpG clock can be found in Suppl. Fig. 1 and Suppl. Fig. 2, respectively. We also find that accurate age estimates are made for samples taken from time points from post-natal mice to mice of advanced age, as can be seen in these figures.

Statistically speaking, the construction of epigenetic clocks is highly degenerate. That is, there are many clocks that select different sites, use slightly different weights, and achieve similar performance. For this reason, we have not emphasized the specific sites used in our clocks, as we do not believe that they are unique, but rather one set among many that could be used to construct clocks. However, we have included two Supplementary Tables of information for two of the clocks. Suppl. Table 1 provides the CpGs used in the elastic net model derived from all available methylation data, along with the regression model coefficients, and the distance of each CpG to the transcription start site of the nearest gene(s). Suppl. Table 2 provides the same information but for the elastic net model derived from the subset of RRBS data corresponding to CpGs that are evolutionarily well-conserved in mammals. For the ridge regression clock based on all CpGs, this information is provided as a text file on the Gene Expression Omnibus (https://www.ncbi.nlm.nih.gov/geo/) under super-series accession number: GSE120137.

**Table 2:** Datasets.

**Reference**	**Tissue**	**Strain**	**Age Dist.**
Novel. Current study	Adipose (56) Blood (72) Kidney (56) Liver (60) Lung (60) Muscle (60)	C57BL/6J (200) BALB/cByJ (164)	N=364 mean = 10.9 median = 10 std dev = 7.4 min = 1.7 max=21.3
Stubbs (2017)	Cortex (16) Heart (15) Liver (15) Lung (15)	C57BL/6	N=61 mean = 5.1 median = 6.21 std dev = 3.4 min = 0.2 max = 9.4
Cole (2017) [45]	Liver (32)	Ames Prop1 Dwarf (16) UM-HET3 (16)	N = 32 mean = 13.5 median = 22 std dev = 9.8 min = 2 max = 22
Petkovich (2017) [44]	Blood (231)	C57BL/6 (161) B6D2F1 (22) GHRKO (26) Snell ([DW/J x C3H/HEJ]/F2) (22)	N = 231 mean = 14.7 median = 9.5 min = 0.6 max = 32.2
Novel. Current study. JAX lab	Kidney (190)	Diversity Outbred (190)	N = 190 mean = 12.1 median = 12 std dev = 4.9 min = 6 max = 18
Reizel (2015) [76]	Cerebellum (8) Liver (49) Muscle (25) Spleen (10)	C57BL/6	N = 92 mean = 2.8 median = 4.6 std dev =1.9 min = 0.23 max = 4.6
Cannon (2016) [67]	Heart (5) Liver (22) Muscle (5)	C57BL/6	N=32 mean = 0.8 median = 0.6 std dev = 0.9 min = -0.6 max = 2.1
Cannon (2014) [52]	Liver (40)	C57BL/6	N = 40 mean = 2.07 std dev = 0 min = 2.07 max = 2.07
Orozco (2014) [66]	Liver (105)	91 different strains	N = 105 mean = 4 median = 4 std dev = 0 min = 4 max = 4

### Diet effects on epigenetic aging

**Figure 2:**
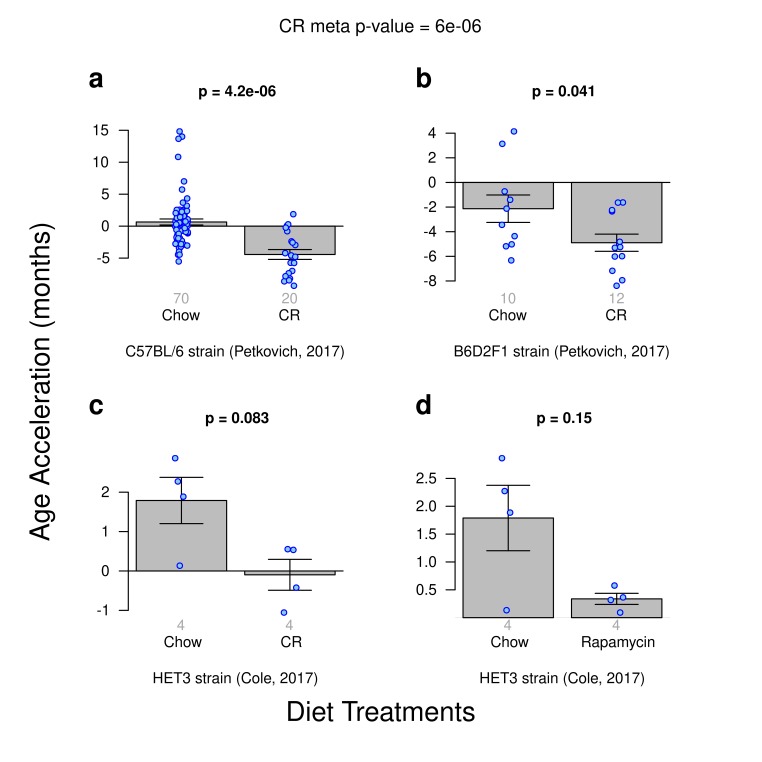
**Age acceleration due to diet treatments.** Results obtained from ridge regression clock. A meta-analysis p-value for the 3 calorie-restriction (CR) experiments is included. (**a**) Calorie restriction versus standard diet in the C57BL/J strain. (**b**) Calorie restriction versus standard chow diet in the B6D2F1 strain. (**c**) Calorie restriction versus standard diet for the HET3 strain. **d**) Rapamycin enriched diet versus standard diet for the HET3 strain.

We analyzed data from 3 calorie restriction experiments and 1 rapamycin diet treatment experiment. These "test data" had been left out of the training set used in the construction of our epigenetic clocks. The most significant results could be observed for the ridge regression clock based on all CpGs (Fig. 2): significantly delayed epigenetic aging effects can be observed in calorie restricted C57BL/6 mice (p=4.2E-6, Fig. 2a) and in B6D2F1 mice (p=0.041, Fig. 2b). A similar pattern could be observed for calorie restricted HET3 mice (Fig. 2c) but the results did not quite reach statistical significance (p=0.083), which might reflect the low sample size (4 CR vs 4 chow fed HET3 mice) or the fact that the latter data had been generated using a different platform (WGBS). However, the age estimates of the WGBS samples (HET3 strain) were consistent with those obtained for RRBS samples despite the absence of WGBS samples from the training set.

CR induced anti-epigenetic aging effects could also be observed with the 2 elastic net clocks (based on all CpGs and on highly conserved CpGs, respectively) but the results were less significant than the above mentioned effects observed for the ridge regression clock (Suppl. Fig. 3).

### No significant effect for rapamycin

The single comparison of mice fed with a rapamycin-enriched diet to those fed a standard diet did not yield a significant difference in age acceleration irrespective of the underlying clock (e.g. p=0.15, Fig. 2d) which might reflect the low sample size (4 Chow vs 4 Rapamycin fed mice) or the fact that the latter data had been generated using a different platform (WGBS).

### Delayed epigenetic aging in dwarf mice

A few transgenic strains of mice have maximum life spans substantially greater than that of most other strains. In particular, the Ames and Snell mice, which have mutations in pituitary transcription factors (and hence are deficient in growth hormones, luteinizing hormone, thyroid-stimulating hormone, and IGF1) have extensions in maximal lifespan of up to 65% [48–50].

Using publicly available data, we aimed to replicate the findings from previous publications on delayed epigenetic aging effects in dwarf mice. Three different experiments within our composite dataset were designed to examine DNA methylation and dwarfism: i) growth hormone receptor knock out mice (GHR-KO) versus wild type mice ([C57BL/6J x BALB/cByJ]/F2), ii) Snell dwarf (SD) mice versus wildtype mice WT ([DW/J x C3H/HEJ]/F2), and iii) Ames dwarf mice versus WT where these were generated by mating either homozygous (df/df) or heterozygous (df/+) dwarf males with heterozygous females (df/+), respectively. The Ames dwarf mouse line carries a recessive mutation in the Prop1 gene and homozygous animals [Prop1(df)/Prop1(df)] show dwarfness and exhibit extended lifespan [51]. Heterozygous littermates [Prop1+/Prop1(df)] were generated by breeding heterozygous females with homozygous males are of normal size.

**Figure 3:**
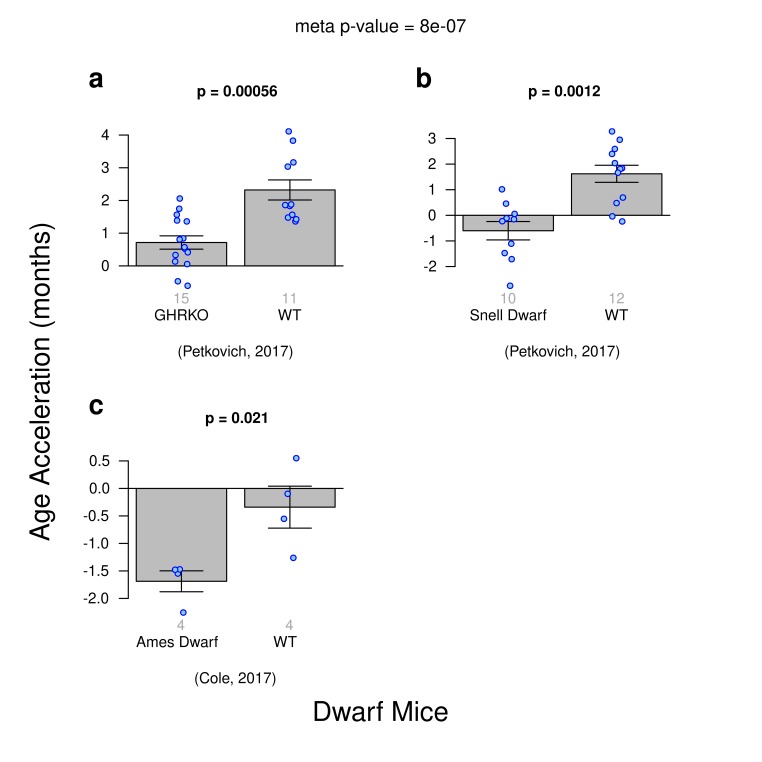
**Age acceleration and Dwarfism in mice.** Results obtained from ridge regression clock. A meta-analysis p-value for the 3 experiments is included. (**a**) Genetic knockout dwarf mice versus wild type. (**b**) Snell dwarf mice versus wild type. **c**) Ames Dwarf mice versus wild type.

The ridge regression clock based on all CpGs managed to detect a delayed-epigenetic aging effect in all three types of dwarf mice (Fig. 3). Despite low sample sizes, the trend for homozygous dwarf strains to slow the epigenetic clock is clear and statistically significant with a meta-analysis p-value of 8E-7. However, these results are not statistically robust with respect to different epigenetic clocks: the association of dwarfism and slow epigenetic aging could *not* be detected with the same significance with the two elastic net regression clocks (Suppl. Fig. 4).

### Maternal diet effects on epigenetic aging

**Figure 4:**
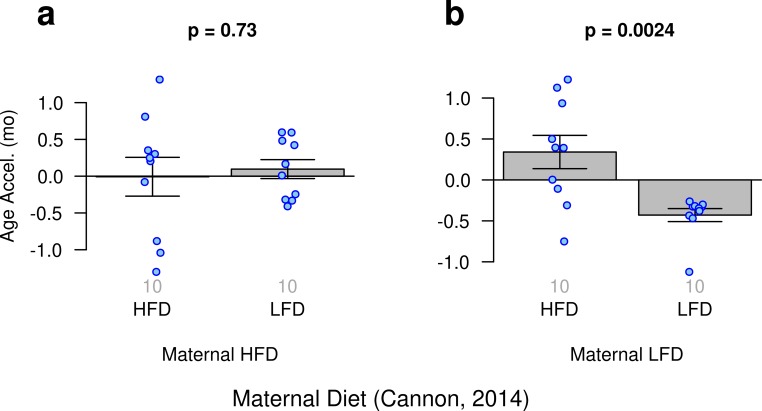
**Age acceleration and maternal diet.** Results obtained from ridge regression clock. (**a**) Offspring of mothers fed a high fat diet (HFD) who were fed either a high fat or low fat diet (LFD). (**b**) Offspring of mothers fed a low fat diet who were fed either a high fat or low fat diet.

Cannon, et al. investigated the potential influence of maternal diet on gene expression and DNA methylation in their offspring [52]. Our ridge regression clock reveals that the slowest epigenetic aging effects can be observed in low fat diet-fed offspring of low fat diet-fed mothers (Fig. 4).

Although, this finding is biologically plausible it is not statistically robust with respect to other epigenetic clocks. In particular, it cannot be observed for the two elastic net regression clocks (Suppl. Fig. 5)

### GWAS of epigenetic age in mice

**Figure 5:**
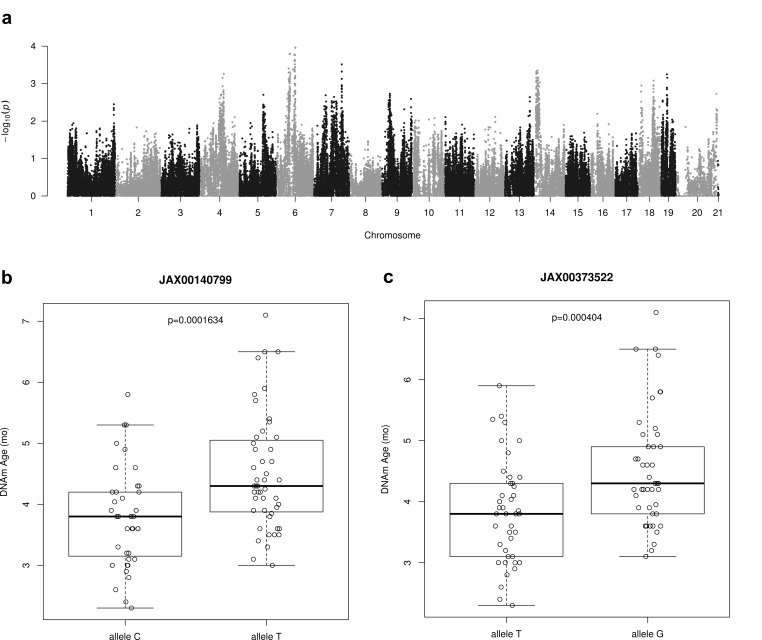
**Genome-wide association results for DNAm Age.** (**a**) Manhattan plot presenting genome-wide association results for DNAm Age. Epigenetic age predictions were calculated using all CpGs clock with ridge regression and leave-one-sample-out estimates. GWAS analysis was based on linear mixed model and a set of 196,148 SNPs (MAF > 0.05) from HMDP mice strains. (**b**) This SNP as identified using GWAS analysis of epigenetic age predictions. It is located in an LD block on chromosome 6 and contains the genes *Npy*, *Mpp6*, *Gsdme* and *Osbp13*. A one-sided t-test of DNAm ages between the two allelic groups shown is statistically significant. (**c**) It is located in an LD block on chromosome 6 and contains the genes *Npy*, *Mpp6*, *Gsdme* and *Osbp13*. A one-sided t-test of DNAm ages between the two allelic groups shown is statistically significant.

We used epigenetic age as trait in our Genome Wide Association Study (GWAS) in the Hybrid Mouse Diversity Panel (HMDP). We calculated epigenetic age via a cross validation approach in order to avoid overfitting. Specifically, epigenetic age was computed for 88 strains using the ridge regression based clock and leaving out from the training set the sample whose age was estimated. The mean calculated epigenetic age was 4.18 months (±0.95) in the range of 2.3-7.1 months with a median of 4.1 months. A set of 196,148 SNPs (MAF>5%) was used for association studies. We used linear mixed models to correct for population structure using the pyLMM software. As our cohort size was limited, we were not able to identify peaks whose significance was beyond the Bonferroni threshold in this analysis (Fig. 5a). Therefore the results presented in Table 3 with the top 10 SNPS are only suggestive of an association and will need to be confirmed in the future with a larger cohort.

**Table 3:** Top ten SNPs from GWAS analysis of DNAm age predictions corresponding to peaks connected to LD blocks in HDMP (~2 Mbp). P-values were computed with a linear mixed-model (LMM).

**SNP ID**	**Chr**	**Position**	**LMM** **p-value**	**Nearest gene(s)**
JAX00189882	6	77104479	1,08E-04	Ctnna2, Lrrtm1
JAX00141186	6	55124351	1,59E-04	Plekha8, Mturn, Znrf2, Nod1, Ggct, Gars, Crhr2, Inmt, Mindy4, Aqp1, Ghrhr, Adcyap1r1
JAX00613802	6	73641278	1,67E-04	Dnah6, Suclg1, 4931417E11Rik
JAX00651898	7	115070069	3,06E-04	Calca, Calcb, Insc, Sox6
JAX00373522	14	14657081	4,48E-04	Olfr720, Olfr31, Il3ra, Slc4a7, Nek10
JAX00049927	14	9498134	4,83E-04	Fhit
JAX00140799	6	49976398	5,06E-04	Npy, Mpp6, Gsdme, Osbp13
JAX00189488	4	95990791	5,48E-04	Fggy, Hook1, Cyp2j13, Cyp2j12, Cyp2j11, Cyp2j8
JAX00087970	19	25717022	5,66E-04	Kank1, Dmrt1, Dmrt3, Dmrt2
JAX00374020	14	17587871	7,02E-04	Thrb

We analysed the genes that were found proximal (500 kbp up- and downstream) to the identified peak SNP site. This list contains several genes that have previously been implicated in aging: *Aqp1* (*Aquaporin 1*) [53], *Npy* (*Neuropeptide Y*) [54], *Adcyap1r1* (*Adenylate cyclase-activating polypeptide type I receptor 1*) as a receptor for PACAP (Pituitary Adenylate Cyclase-Activating Peptide) [55,56]. The most notable is *Npy*, which encodes a hormone responsible for appetite control [57], regulation of fat metabolism [58], and plays a critical role in caloric restriction (CR) mediated lifespan extension [59]. As examples, we found statistically significant differences (p < 0.001) in average DNAm ages of mice strains having allele C or T within SNP JAX00140799 (Fig. 5b) and between mice strains having allele T or C within SNP JAX00373522 (Fig. 5c).

## DISCUSSION

Based on multiple tissue samples taken from previous studies and our own in-house collection we compiled a dataset of 1189 mouse DNA methylation measurements across hundreds of thousands of CpGs. These samples represent the most comprehensive dataset thus far of matched single base resolution methylomes in mice across multiple tissues and ages.

We demonstrate that these data enable construction of highly accurate multi-tissue age estimation methods (epigenetic clocks) for mice that apply to the entire life course (from birth to old age). We demonstrate that these clocks perform well on new tissues not included in the training of the clock by performing tissue exclusion cross-validation. This gives us confidence that these clocks will work on new samples from other tissue types as well. However, we cannot rule out that these clocks fail in specific cell types. Epigenetic age estimators that focus on specific tissues or cell types can have greater accuracy than pan tissue age estimators [60].

Our study leads to several novel insights. First, our first prototype of an age estimator based on fewer than 1000 highly conserved CpGs demonstrates that it will be feasible to build highly accurate DNAm age estimator on the basis of highly conserved CpGs.

Second, we find that epigenetic clocks that are optimal for estimating age (namely those based on elastic net regression) may be inferior to less accurate clocks (based on ridge regression) when it comes to gold standard anti-aging interventions. Only our ridge regression clock manages to corroborate most of the previously reported findings, *e.g.* only the ridge clock showed that dwarf strains show slower epigenetic aging relative to wild-type strains. The anti-epigenetic aging effects of calorie restriction are highly robust and could be observed with all clocks. Moreover, by utilizing epigenetic ages as phenotypic traits in a GWAS study of 88 strains of mice we found suggestive associations with several genes, including neuropeptide Y whose role in appetite control and calorie restriction mediated lifespan extension is well documented. However, none of our clocks managed to detect an anti-aging effect of rapamycin in a small data set which might reflect the low sample sizes or technical reasons including low coverage afforded by the measurement platform (WGBS).

All clocks were able to detect a slowing of the epigenetic clock in mice fed a calorie restricted diet, though with differing sensitivity, suggesting that the effects of calorie restriction are pervasive across the methylomes. In contrast, the slowing of epigenetic aging in mice fed a low fat diet for two generations were not detected by the clocks with fewer CpGs (elastic net), suggesting these effects are either more subtle or more localized in the methylome.

These results suggest that the multi-tissue ridge regression DNA-methylation clock is most useful in assessing “biological age” for a variety of treatments, experimental interventions, and genetic backgrounds. However, the elastic net clocks are better for assessing chronological age. We evaluated both ridge and lasso regression in previous studies with human data and found that lasso outperformed ridge not just in terms of accuracy but also in terms of interpretability (unreported findings). Therefore, it is a curious finding that ridge regression has some merits when it comes to mice.

We speculate that one reason that ridge regression works best in our context is that our dataset is more heterogeneous than those used in previous studies. Our dataset includes mice of different ages, strains and diverse tissues, all collected in different labs and resulting in a whole that is larger than any previous dataset. Because genetic diversity in mice is high, it is possible that lasso models that only use a limited number of sites are more prone to be influence by genetic variation (as DNA methylation is often associated with genetic variation). Thus, on the whole, it is possible that the ridge approach minimizes these effects by using all sites, and thus leads to the most robust overall performance.

The DNAm age estimates from our mouse clocks exhibit a correlation coefficient with chronological age that ranges from R=0.79 to 0.89 (Table 1). These correlations are only slightly weaker than those observed for human studies (R=0.96 for the pan tissue estimator from Horvath). We have no doubt that more accurate mouse clocks can be built by reducing technical variation and by employing even larger data sets.

We acknowledge several limitations. Our genetic study of epigenetic aging rates was under-powered. Large scale *human* studies have implicated genome-wide significant loci including the *TERT* gene [61–63]. We did not assess the intra-assay variation of replicate samples in the current article but refer the interested reader to relevant articles [59,60].

Rigorous quantitative comparisons to previously published mouse clocks (*e.g.* comparing age correlation values) could not be made due to technical differences in the processing of sequence data, in the estimation and correction of methylation calls, in the limited tissue sampling of previous clocks, and, most importantly, in the simple absence of particular CpGs in the various datasets and clock models. The subset of CpGs with high coverage in one RRBS data set tends to exhibit poor overlap with a subset of CpGs from another RRBS data set. This poor overlap of CpGs makes it difficult to validate epigenetic clocks based on RRBS data. We are currently working on a custom methylation array platform that avoids these pitfalls.

## METHODS

### Data sets

We generated reduced representation bisulfite sequencing **(**RRBS) methylation data for mouse adipose, blood, liver, and kidney, muscle, and lung tissue samples using the protocol below.

### DNA methylation assay

Genomic DNA was isolated by standard phenol-chloroform extraction method and used as input to prepare Reduced Representation Bisulfite Sequencing (RRBS) libraries as described previously [64] with minor modifications. For each sample 50-100 ng of purified genomic DNA was digested with 20 U of MspI (NEB, cat # R0106L) at 37°C o/n in the presence of RNase Cocktail Mix (Ambion, cat # AM2286). End-repair and dA-tailing was performed by the addition of Klenow Fragment 3’->5’ exo- (NEB, cat # M0212L) in the presence of dATP, dGTP and d5mCTP (Fermentas). Adapter Ligation was performed by the addition of 0.3 µl of Illumina TruSeq methylated Adapters (Illumina, TruSeq Nano cat# FC-121-4001) and 2 µl of Illumina Ligation Mix 2 (Illumina, TruSeq Nano cat# FC-121-4001). Samples were pooled and purified using an equal volume of SPRI beads (Beckman Coulter, cat # B23318). Size-selection was performed using SPRI beads to enrich for fragments from 200 to 300 bp. Bisulfite treatment was performed using Epitect Bisulfite kit (QIAGEN, cat # 59104) according to manufacturer's protocol, except that two consecutive rounds of conversion are performed, for a total of 10 hr of incubation. Purified converted DNA was PCR amplified using MyTaq HS Mix (Bioline, cat# BIO-25045) and TruSeq PCR Primer Cocktail (Illumina, TruSeq Nano cat# FC-121-4001) according to the following protocol: initial denaturation at 98°C for 30s; 12 cycles of 98°C for 15s, 60°C for 30s, 72°C for 30s; final extension at 72°C for 5 min. Amplified libraries were purified twice with an equal amount of SPRI beads to remove primer and adapter dimers. Libraries were sequenced 100 bp single-end on an Illumina HiSeq4000. For the kidney data from JAX laboratories, the sequencing protocol was as follows. RRBS libraries were prepared using 100 ng DNA, the Ovation RRBS Methyl-Seq System 1–16 (NuGEN Technologies, San Carlo, CA) part number 0353, and the EpiTect Fast 96 Bisulfite Conversion kit (Qiagen, Hilden, Germany) part number 59720. The manufacturer’s protocols were followed except for the number of PCR cycles in the library amplification step, which was increased, from 12 to 13. Libraries were quantified using the Library Quantification Kit (Kapa Biosystems, Wilmington, MA) part number KK4835, normalized to 10nM, and pooled in groups of 12. Each pool was sequenced 1 x 100 bp on one lane of the HiSeq2500 (Illumina, San Diego, CA) at The New York Genome Center (New York, NY).

These datasets were integrated with RRBS data made available to the public via the GEO repository [65]. We included datasets from previous RRBS-based “epigenetic clock” studies [44,47] along with RRBS data from an EWAS study of metabolic traits [66], from a study of maternal diet effects on gene expression and DNA methylation [52], from a study of post-natal hepatocyte development [67], from a multi-tissue study of sex hormone effects on DNA methylation [68].

### Kidney data from the Jackson Laboratory

Kidneys were collected from male and female Diversity Outbred mice at ages 6, 12 and 18 months. Mice were group housed in SPF condition and fed a standard lab chow diet (5K0G) with 6% calories from fat. Tissues were flash frozen in LN2, pulverized and mixed prior to DNA extraction. RRBS sequencing was carried out at the New York Genome Center. Although whole-genome bisulfite-sequencing data (WGBS) has a variety of characteristic differences from RRBS, we obtained the set of this data collected previously to examine longevity interventions in mice and build an epigenetic clock [45,46].

### Data processing

Where possible, we downloaded the raw sequencing files from previous studies via (GEO) and performed alignments and methylation calling identically as for our in-house data using BS_Seeker2 with default parameters [69]. All mouse methylation data in this study utilized mouse genome mm10 coordinates. When technical replicates were available for a given sample, they were merged by summing the sequencing counts.

For the kidney data from JAX laboratories, a Bismark-based pipeline was initially used as follows [70]. All the samples were subjected to QC using the trim_galore module then trimming of the diversity adapters was performed by trimRRBSdiversityAdaptCustomers.py script from NuGen. High quality trimmed reads were aligned to all eight diversity outbred founder strains (A/J, C57BL/6J, 129S1/SvImJ, NOD/ShiLtJ, NZO/HlLtJ, CAST/EiJ, PWK/PhJ, and WSB/EiJ,) separately using Bismark at default parameters. The alignment to founder genomes except C57BL/6J were converted to reference genome coordinates (mm10) using g2gtools v1. Then, we selected the reads which were mapped to the same locus in multiple founders strains (assigned to founder strains with minimum edit distance) or mapped uniquely to one founder strains by custom in-house script. The bed file of estimated methylation proportion of each founder (except C57BL/6J ) was converted to reference genome coordinate by g2gtools v1 and, finally, combined to provide the methylation proportion in each diversity outbred animals.

For each CpG site in each sample we estimated the methylation frequency as the number of methylated mapped read counts over the total mapped read counts. Where available, the counts for the forward and reverse Cytosines of the CpG were pooled and treated as a single measurement. We then computed a 95% confidence interval with a Bayesian approach using a Beta distribution (0.5,0.5) (“Jeffrey’s Prior”) for all methylation values [71, 72]. For inclusion in our analysis, we required that each CpG site had confident methylation frequencies in at least 95% of samples. Confidence was defined as having a confidence interval smaller than 0.50. True missing values or measurements failing that confidence interval filter were imputed using k-nearest-neighbor approach with k=5. This select strategy resulted in CpGs whose mean methylation levels ranged from zero to 1 (Suppl. Fig. 6).

### Sample exclusion

In order to maximize the number of samples and coverage of the methylomes, it was necessary to exclude a number of samples both from our new data and from previously published datasets. First, we removed samples with fewer than 500,000 measured CpGs. Next, after an initial matrix was constructed, we iteratively removed samples with the most missing values until we arrived at a matrix with ~2% total missing values.

### Penalized regression models

Penalized regression models were created with glmnet [73]. We investigated models produced by both elastic net regression (alpha=0.5) and ridge regression (alpha=0). The optimal penalty parameters in all cases were determined automatically by using a 10 fold internal cross-validation (cv.glmnet) on the training set. By definition, the alpha value for the elastic net regression was set to 0.5 (midpoint between ridge and lasso type regression) and was not optimized for model performance. We omitted the results from lasso regression models (alpha=1) because the age estimates tended to be less accurate than those from elastic net regression.

The covariates in our data (methylation of CpGs) are known to have a high degree of multicollinearity. While lasso and elastic net regression allow regression coefficients to go to zero and thus yield a sort of “feature selection” which is desirable for interpretability, the correlations among methylation sites may contain subtle information that might be useful to retain (which supports the use of ridge regression).

### Cross-validation estimates of accuracy

We performed three types of cross-validation schemes for arriving at unbiased (or at least less biased) estimates of the accuracy of the different DNAm based age estimators. One type consisted of leaving out a single sample (LOOCV) from the regression, predicting an age for that sample, and iterating over all samples. The second type (10-fold) was similar to the first except that 10% of samples were withheld per iteration. The third type consisted of iteratively leaving out all samples of a particular “batch” where batched was defined as combination of tissue type and publication of origin. For example, three batches resulted from a single publication if the underlying RRBS data were obtained from 3 distinct tissues. Samples from longevity intervention studies (CR, Rapamycin, dwarf mice) along with their respective control samples were excluded from all instances of training-set construction.

### Conserved-CpG clock

Our team is currently developing a mammalian DNA-methylation array for measuring methylation levels in mammals. The primary selection criteria for CpGs for the probe-design of this bead-chip array was conservation of the local sequence context of the CpG across 60 sequenced mammalian genomes. We obtained the list of candidate CpGs in this effort and intersected it with CpGs in our high-quality RRBS data.

### Epigenetic age acceleration

To investigate effects of biological interventions and genetic background on epigenetic aging, we employ a quantity termed “age acceleration”. In the simplest form, it is just the difference between the epigenetic age estimated by the clock and the chronological age. However, this measure can be age-dependent itself, causing difficulty in interpretation. Instead, age acceleration is computed as the residual, per sample, after fitting predicted ages to chronological ages. This fitting is done on a per-batch basis. P-values for age acceleration comparisons found in Figs 2, 3, 4, and Suppl. Figs 3, 4 and 5 were obtained using the non-parametric Kruskal-Wallis test.

### Genome-wide association study

GWAS was performed using 88 strains from the Hybrid Mouse Diversity Panel (HMDP, listed in Suppl. Table 1), that have been extensively used as a resource for systems genetics analyses [66,74,75]. Of the total of 459 895 SNPs, we selected a set of 196 148 SNPs that exhibited minor allele frequency greater than 5%. DNAm ages, computed using all CpGs clock with ridge regression and leave-one-sample-out estimates, were treated as phenotypes in the association studies. All of the mice were at chronological age of 4 months. GWAS was conducted using the linear mixed model python package pyLMM to account for population structure and relatedness among the mouse strains. We selected the top SNPs in each of the peaks from the pyLMM analysis that were located a minimum of 2 Mbp apart, which is the average of LD block size for SNPs in the HMDP.

### Data availability

Raw sequencing data and processed data for samples collected at UCLA and JAX, as well as re-processed data from previous studies have been made available at the Gene Expression Omnibus (https://www.ncbi.nlm.nih.gov/geo/) under super-series accession number: GSE120137

## Supplementary Material

Supplementary Figures

Supplementary Table 1

Supplementary Table 2

Supplementary Table 3
