# Supplementary material for: A multi-tissue full lifespan epigenetic clock for mice
Source: Aging (Albany NY). 2018 Oct 21;10(10):2832–54. doi: 10.18632/aging.101590 (PMC6224226; doi:10.18632/aging.101590)
Supplement: Supplementary Figures [file aging-10-101590-s001.pdf]

SUPPLEMENTARY MATERIAL

This information is provided as supporting information and results to the main ones presented in the manuscript.

SUPPLEMENTARY FIGURES

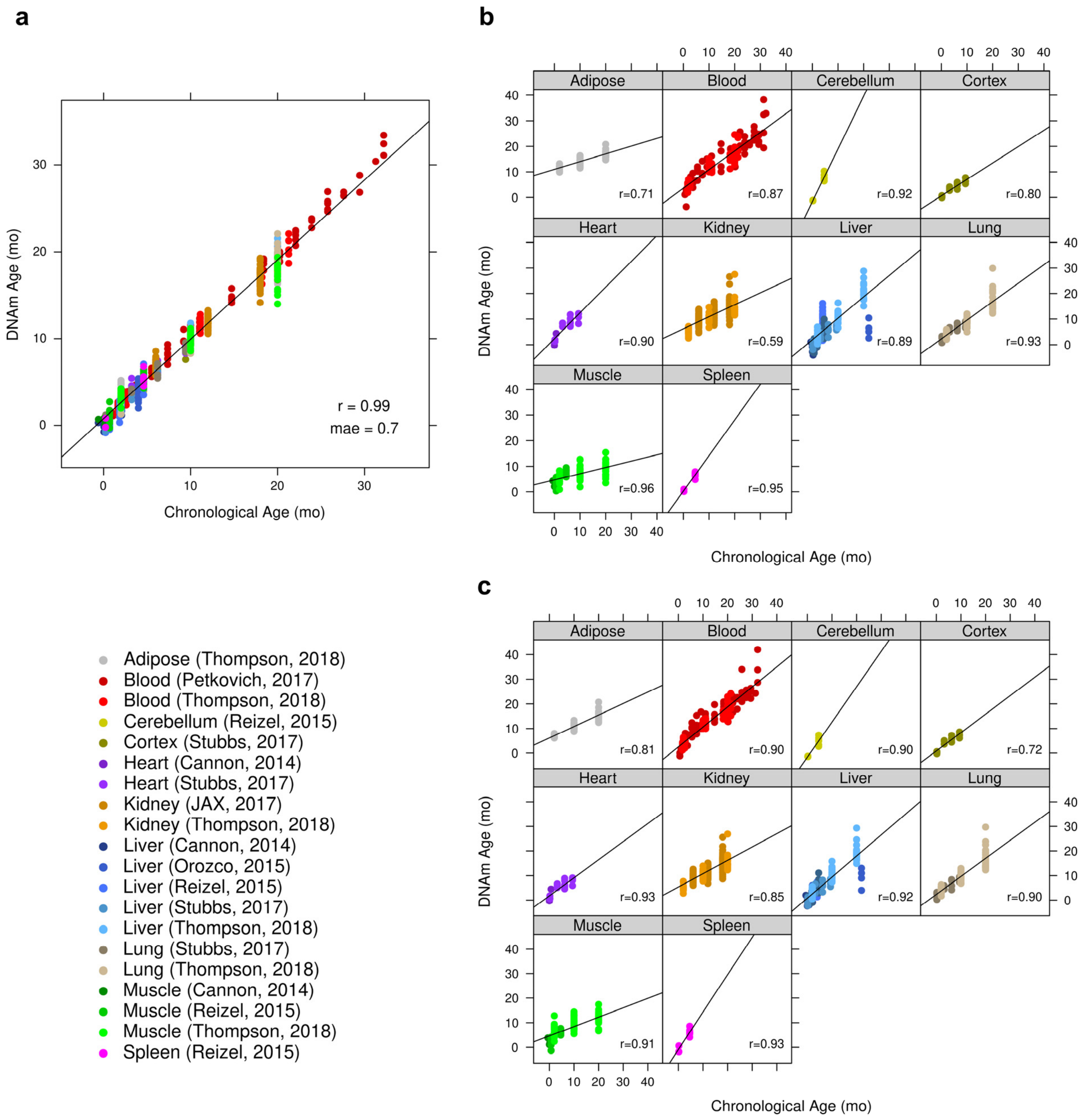

Supplementary Figure 1. Accuracy of the elastic net clock based on all CpGs. DNA methylation age (y-axis) versus chronological age (x-axis) for all mouse samples. (**a**) Performance of elastic regression clock on all training samples. (**b**) Results by tissue type of cross-validated predictions obtained by iteratively withholding one “batch” (tissue x publication). (**c**) Results by tissue type of predictions from leave-one-out-cross-validation.

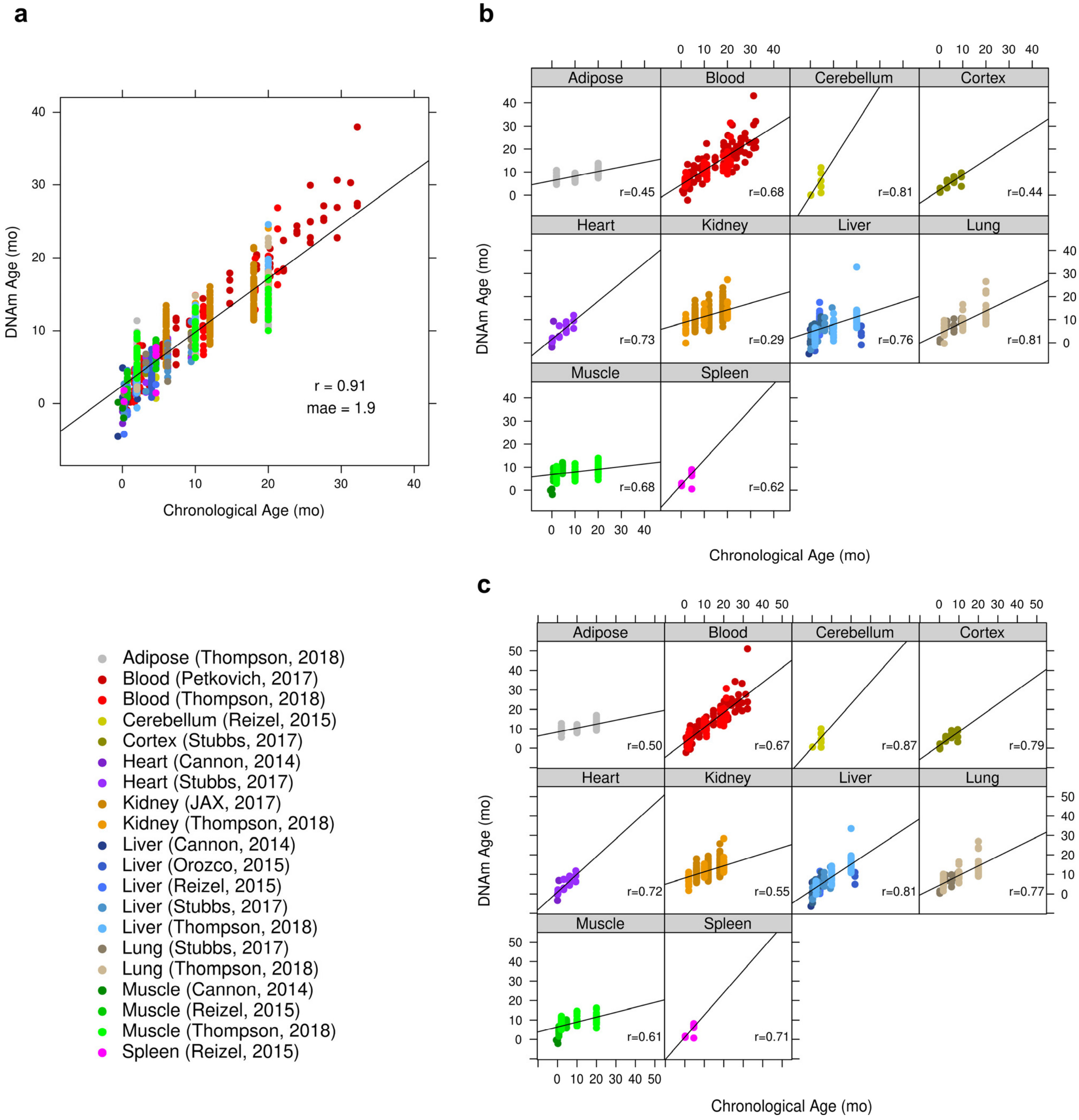

Supplementary Figure 2. Accuracy of the conserved clock based on elastic net regression. DNA methylation age (y-axis) versus chronological age (x-axis) for all mouse samples. (**a**) Performance of elastic net regression clock on all training samples. (**b**) Results by tissue type of cross-validated predictions obtained by iteratively withholding one "batch" (tissue x publication). (**c**) Results by tissue type of predictions from leave-one-out-cross-validation.

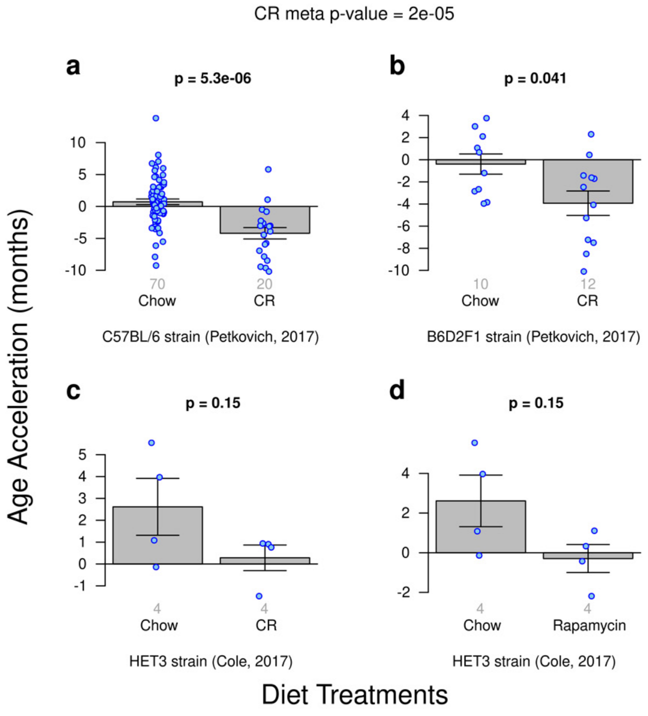

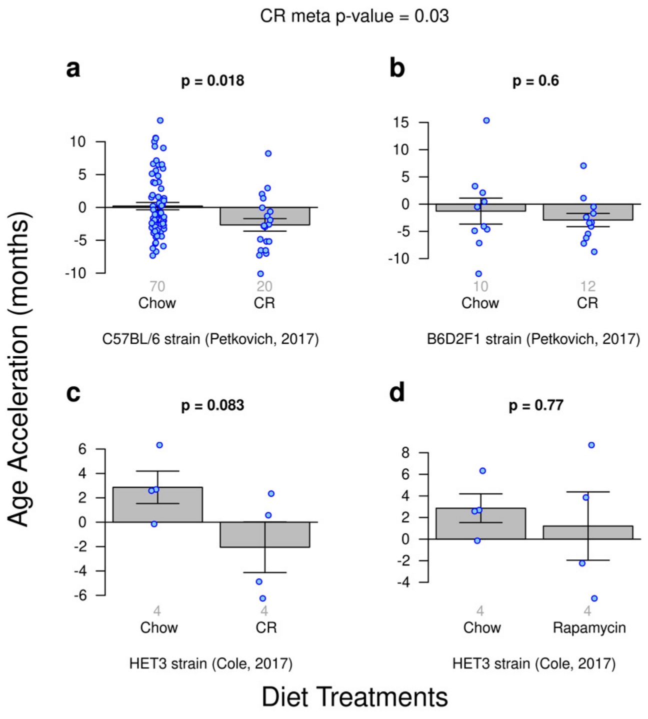

Supplementary Figure 3. This figure corresponds to Fig. 2 in the main text for diet treatments and longevity. The upper panel shows the same results for an elastic net clock using all CpGs as input. The lower panel shows the same results for an elastic net clock using only conserved CpGs as input. Overall, the results for these two clocks are less significant than those observed for the ridge regression clock. But both of these clocks detect anti-epigenetic aging effects CR in the C57BL/6 strain (panel a).

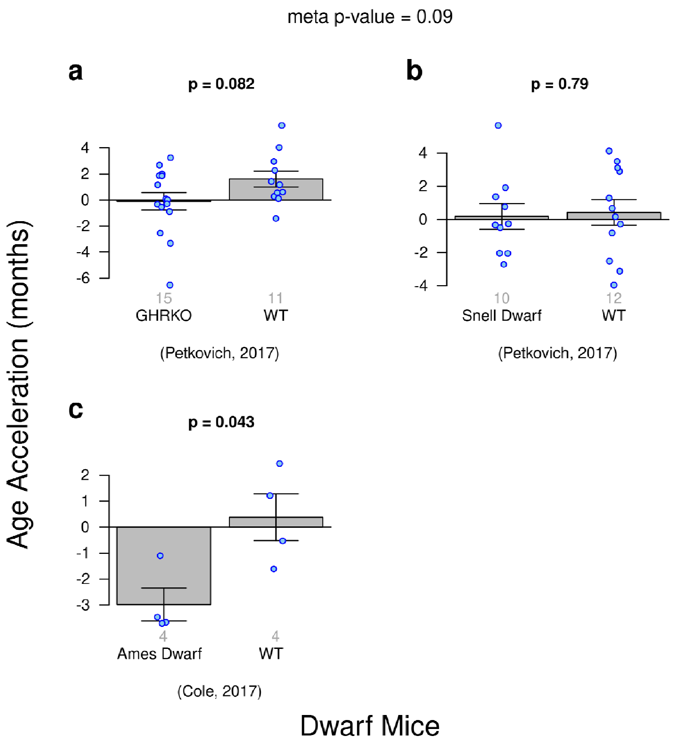

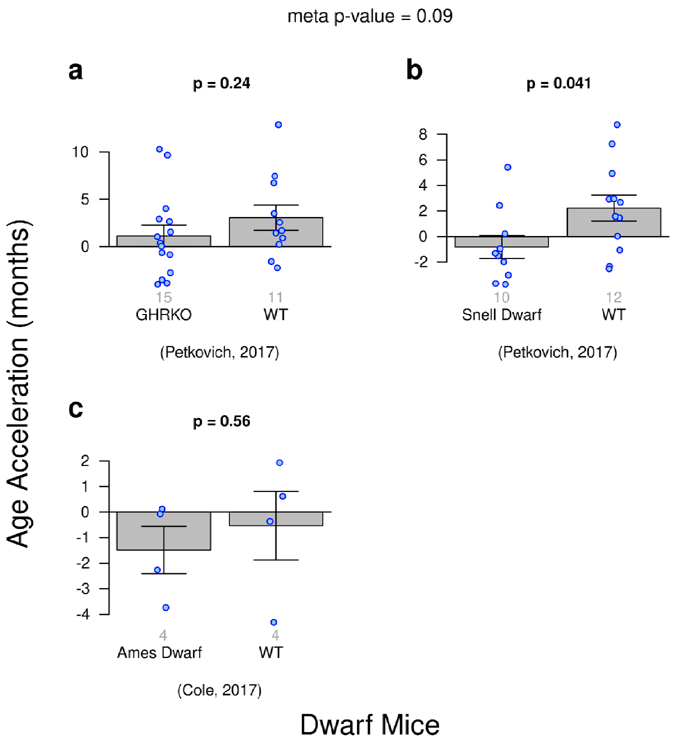

Supplementary Figure 4. This figure corresponds to Fig. 3 in the main text for dwarfism and longevity. The upper panel shows the same results for an elastic net clock using all CpGs as input. The lower panel shows the same results for an elastic net clock using only conserved CpGs as input.

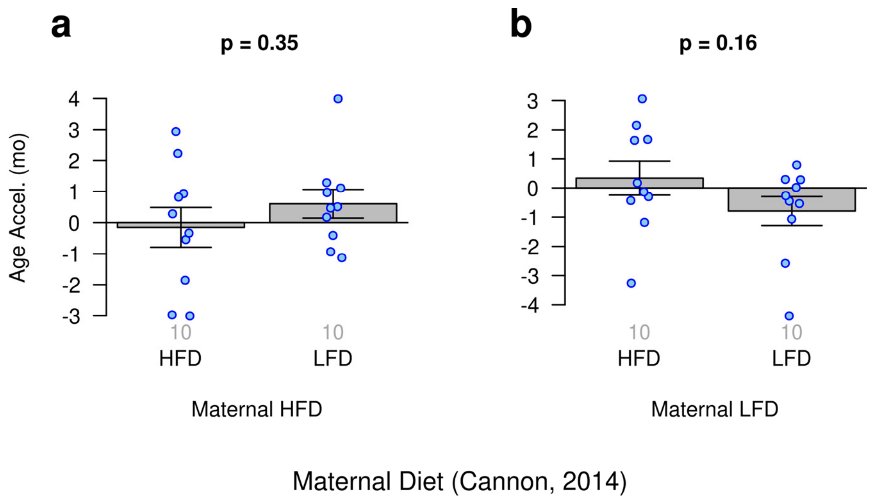

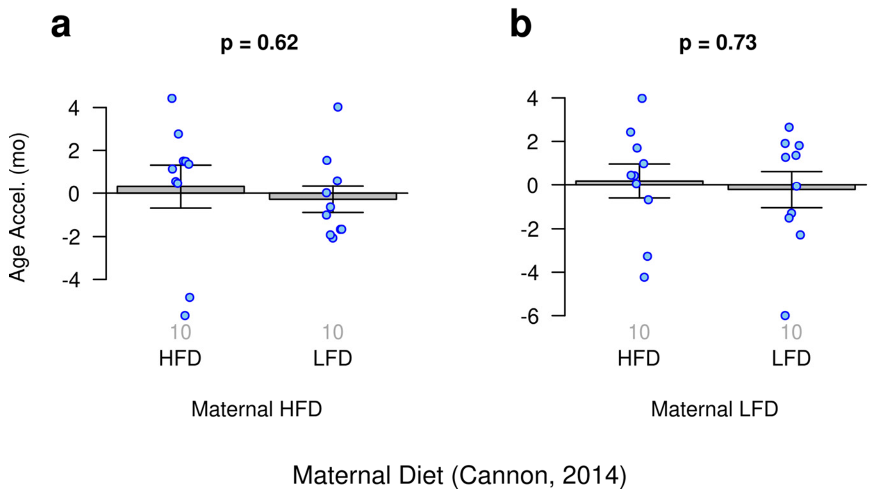

Supplementary Figure 5. This figure corresponds to Fig. 4 in the main text for the effects of maternal diet on DNA methylation in offspring. The upper panel shows the same results for an elastic net clock using all CpGs as input. The lower panel shows the same results for an elastic net clock using only conserved CpGs as input**.**

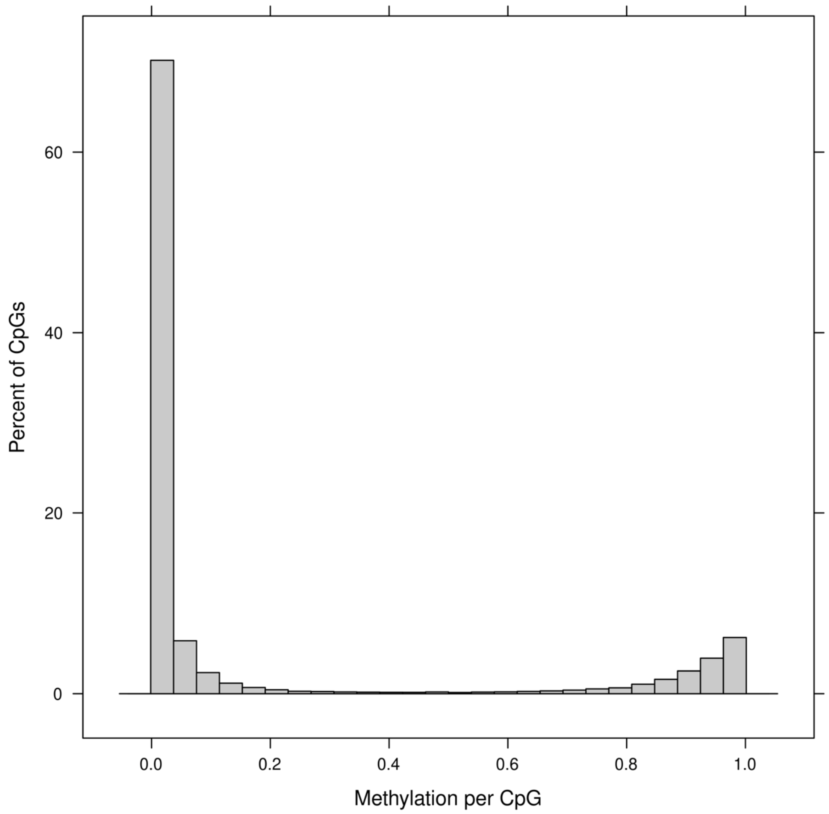

Supplementary Figure 6. Histogram of methylation levels of all CpGs in the training set of samples.
