# Supplementary material for: A multi-tissue full lifespan epigenetic clock for mice
Source: Aging (Albany NY). 2018 Oct 21;10(10):2832–54. doi: 10.18632/aging.101590 (PMC6224226; doi:10.18632/aging.101590)
Supplement: Supplementary Table 1 [file aging-10-101590-s002.docx]

**Supplementary Table 1.** CpGs utilized in the elastic net age clock derived from all CpGs measured. Listed are the genomic coordinates, the linear coefficients of the model, and the distances to the transcription site (TSS) of the nearest genes.

| **Chromosome** | **Coordinate** | **Coefficient** | **Genes (distance to TSS)** |
| --- | --- | --- | --- |
| Intercept |  | 30.3172 |  |
| chr1 | 9967422 | 0.7605 | Gm10567 (-4614) |
| chr1 | 9967428 | 0.1261 | Gm10567 (-4620) |
| chr1 | 13126576 | 4.3766 | Prdm14 (+586) |
| chr1 | 15286937 | 5.6856 | Kcnb2 (-321) |
| chr1 | 46800385 | -0.5194 | Slc39a10 (+53660) |
| chr1 | 55087536 | -0.6355 | Hspe1 (-595), Hspd1 (+487) |
| chr1 | 63273286 | 0.4166 | Zdbf2 (+22) |
| chr1 | 63273288 | 1.2926 | Zdbf2 (+24) |
| chr1 | 64690282 | 5.5767 | Ccnyl1 (-1062) |
| chr1 | 71603645 | -0.9544 | Apol7d (-49191), Atic (+46496) |
| chr1 | 79761546 | -5.0494 | Wdfy1 (+222) |
| chr1 | 79858375 | -0.2711 | Serpine2 (+320) |
| chr1 | 84695279 | 0.2488 | Dner (+941) |
| chr1 | 84934769 | -0.8901 | Slc16a14 (+355) |
| chr1 | 89455649 | 0.7444 | Agap1 (+839) |
| chr1 | 92848744 | 0.0392 | Gpc1 (+17100), Ankmy1 (+54161) |
| chr1 | 92848748 | 0.4056 | Gpc1 (+17104), Ankmy1 (+54157) |
| chr1 | 95666316 | -2.0561 | St8sia4 (+1277) |
| chr1 | 103479589 | 0.5519 | NONE |
| chr1 | 105270925 | -0.6631 | Rnf152 (+85784) |
| chr1 | 106758726 | -0.8413 | Bcl2 (-44453), Kdsr (+1015) |
| chr1 | 118310888 | -3.922 | Tsn (+243) |
| chr1 | 118311288 | -3.1032 | Tsn (-157) |
| chr1 | 119648512 | -1.9629 | Epb4.1l5 (+487) |
| chr1 | 120602265 | 2.8247 | En1 (-152) |
| chr1 | 120602331 | -0.3255 | En1 (-86) |
| chr1 | 128359556 | 1.7188 | Mcm6 (+99) |
| chr1 | 132331689 | -1.5644 | Nuak2 (+15564), Tmcc2 (+59591) |
| chr1 | 132937328 | -0.7063 | Lrrn2 (+57056), Mdm4 (+88019) |
| chr1 | 135374481 | -2.196 | Shisa4 (+755) |
| chr1 | 151345609 | 2.9407 | Ivns1abp (+1112), Swt1 (+82845) |
| chr1 | 152766494 | -1.279 | Arpc5 (-47) |
| chr1 | 163779885 | -2.1693 | Kifap3 (+303) |
| chr1 | 166254097 | 0.2376 | Ildr2 (-41) |
| chr1 | 167078000 | 1.5658 | Fam78b (+76584) |
| chr1 | 180162337 | -0.1488 | Adck3 (+33682) |
| chr1 | 181143980 | -3.359 | Nvl (+176) |
| chr1 | 193130048 | 7.7219 | Diexf (+202) |
| chr2  www.aging-us.com 1 AGING | 18688874 | 1.1456 | BC061194 (-10148), Bmi1 (+11857) |
| chr2 | 18688890 | 2.4924 | BC061194 (-10132), Bmi1 (+11873) |
| chr2 | 18688999 | 2.7974 | BC061194 (-10023), Bmi1 (+11982) |
| chr2 | 18689011 | 0.0009 | BC061194 (-10011), Bmi1 (+11994) |
| chr2 | 18689050 | 3.5852 | BC061194 (-9972), Bmi1 (+12033) |
| chr2 | 18689075 | 8.0881 | BC061194 (-9947), Bmi1 (+12058) |
| chr2 | 19658357 | -4.2881 | Otud1 (+296) |
| chr2 | 24367551 | 0.0149 | Psd4 (-58) |
| chr2 | 26139935 | -1.2542 | Nacc2 (-47759), Lhx3 (+66639) |
| chr2 | 29206237 | 1.1064 | Ntng2 (+41802), Setx (+81246) |
| chr2 | 29540277 | -0.8333 | Rapgef1 (-79442) |
| chr2 | 29803634 | -0.2422 | Urm1 (-23707), Slc27a4 (+1001) |
| chr2 | 31314497 | 2.7303 | Hmcn2 (+83) |
| chr2 | 31383687 | -0.0353 | Ass1 (-86519), Hmcn2 (+69273) |
| chr2 | 32258282 | -1.1623 | Uck1 (+1876), Pomt1 (+21693) |
| chr2 | 32534814 | -0.4353 | Fam102a (-544) |
| chr2 | 32583296 | -0.3936 | St6galnac4 (-3798) |
| chr2 | 34371824 | -1.0745 | Pbx3 (+425) |
| chr2 | 65753979 | 1.2548 | Csrnp3 (-91787) |
| chr2 | 71545678 | 1.233 | Dlx2 (+1075), Dlx1 (+17566) |
| chr2 | 73271897 | 3.9804 | Sp9 (-27) |
| chr2 | 73271967 | 0.0034 | Sp9 (+43) |
| chr2 | 73271991 | 2.0252 | Sp9 (+67) |
| chr2 | 73271993 | 1.3904 | Sp9 (+69) |
| chr2 | 74655567 | 2.0643 | Lnp (-76620), Hoxd10 (-35522), Hoxd11 (-26431), Hoxd12 (-19361), Hoxd13 (-12742), Evx2 (+3851) |
| chr2 | 74655652 | 0.7246 | Lnp (-76705), Hoxd10 (-35437), Hoxd11 (-26346), Hoxd12 (-19276), Hoxd13 (-12657), Evx2 (+3766) |
| chr2 | 74655663 | 0.6198 | Lnp (-76716), Hoxd10 (-35426), Hoxd11 (-26335), Hoxd12 (-19265), Hoxd13 (-12646), Evx2 (+3755) |
| chr2 | 74656282 | 0.0691 | Lnp (-77335), Hoxd10 (-34807), Hoxd11 (-25716), Hoxd12 (-18646), Hoxd13 (-12027), Evx2 (+3136) |
| chr2 | 74657635 | 3.7479 | Lnp (-78688), Hoxd10 (-33454), Hoxd11 (-24363), Hoxd12 (-17293), Hoxd13 (-10674), Evx2 (+1783) |
| chr2 | 74682177 | 0.1683 | Hoxd10 (-8912), Hoxd11 (+179), Hoxd13 (+13868) |
| chr2 | 74763563 | 0.0646 | Hoxd1 (+584), Hoxd13 (+95254) |
| chr2 | 83724981 | -0.0784 | Itgav (+585) |
| chr2 | 90580725 | -0.0283 | Ptprj (-79) |
| chr2 | 92055426 | 0.3096 | Creb3l1 (-30925) |
| chr2 | 93195765 | 0.2594 | Trp53i11 (+8218) |
| chr2 | 102452235 | -0.0148 | Fjx1 (-444) |
| chr2 | 103797471 | -1.2176 | Caprin1 (+177) |
| chr2 | 109676136 | 2.7676 | Bdnf (+1437) |
| chr2 | 109792568 | 0.3912 | Lin7c (-98284) |
| chr2 | 116535471 | 0.4174 | NONE |
| chr2 | 120039017 | -0.8105 | Pla2g4b (+5585), Sptbn5 (+18479) |
| chr2 | 126780509 | 0.4872 | Gm10774 (-70943), Usp50 (+2934) |
| chr2  www.aging-us.com 2 AGING | 127363004 | 0.6359 | Adra2b (-281) |
| chr2 | 128794537 | -0.0866 | Tmem87b (-23580), Mertk (+95582) |
| chr2 | 128967551 | -1.1529 | Zc3h6 (+150), Gm10762 (+173) |
| chr2 | 131179262 | -2.3901 | Spef1 (-4062), Cenpb (+749) |
| chr2 | 131262494 | -0.2245 | Pank2 (-5) |
| chr2 | 132219269 | -0.5242 | Slc23a2 (-74162), Tmem230 (+28384) |
| chr2 | 143862610 | 0.1244 | Bfsp1 (+562) |
| chr2 | 147083923 | 0.702 | Nkx2-4 (+1521), Xrn2 (+70928) |
| chr2 | 147084014 | 1.8955 | Nkx2-4 (+1430), Xrn2 (+71019) |
| chr2 | 147084073 | 1.2298 | Nkx2-4 (+1371), Xrn2 (+71078) |
| chr2 | 147084603 | 1.2617 | Nkx2-4 (+841) |
| chr2 | 147084610 | 2.3309 | Nkx2-4 (+834) |
| chr2 | 147085213 | 1.856 | Nkx2-4 (+231) |
| chr2 | 147365634 | 0.7749 | Pax1 (+641) |
| chr2 | 152414129 | 0.916 | 6820408C15Rik (-1457), Zcchc3 (+914) |
| chr2 | 152943653 | 0.5674 | Foxs1 (-10446), Dusp15 (+8044) |
| chr2 | 152943674 | 1.8634 | Foxs1 (-10467), Dusp15 (+8023) |
| chr2 | 154372729 | -2.4023 | Cdk5rap1 (+280) |
| chr2 | 155956842 | -3.446 | Cep250 (+285) |
| chr2 | 158117908 | 0.1537 | Tgm2 (+28527), Rprd1b (+89412) |
| chr2 | 164164697 | -1.7677 | Kcns1 (+6415), Stk4 (+90560) |
| chr2 | 165116262 | 0.1938 | 1700025C18Rik (-25513) |
| chr2 | 165821819 | -0.31 | Zmynd8 (+36353) |
| chr2 | 173276460 | 1.2198 | Pmepa1 (+72) |
| chr2 | 173565625 | -0.1764 | Ankrd60 (+12739), 1700021F07Rik (+43040) |
| chr2 | 174464089 | 4.523 | Atp5e (+15) |
| chr2 | 180524878 | 0.3324 | Mrgbp (-57833), Ntsr1 (+24903) |
| chr2 | 180702060 | 2.8941 | Dido1 (+289) |
| chr2 | 180776945 | -1.7037 | Bhlhe23 (-46) |
| chr3 | 28805649 | -0.0612 | Rpl22l1 (+214) |
| chr3 | 40541009 | 2.7445 | Intu (+243) |
| chr3 | 51484124 | 2.2116 | Rab33b (+159) |
| chr3 | 53017230 | -1.4665 | Cog6 (-8) |
| chr3 | 53452419 | -0.8884 | Nhlrc3 (+10871) |
| chr3 | 58692509 | 6.6892 | Siah2 (-110) |
| chr3 | 66979873 | 0.0221 | Shox2 (+1897) |
| chr3 | 69316410 | -0.5824 | Ppm1l (-450) |
| chr3 | 69316712 | -1.3507 | Ppm1l (-148) |
| chr3 | 88332469 | -0.0444 | Smg5 (-3790), Tmem79 (+2034) |
| chr3 | 89764409 | 1.8157 | Chrnb2 (+222) |
| chr3 | 89764422 | 6.2341 | Chrnb2 (+209) |
| chr3 | 89764447 | 1.4041 | Chrnb2 (+184) |
| chr3 | 89764485 | 1.0572 | Chrnb2 (+146) |
| chr3 | 91930033 | 0.2753 | Pglyrp3 (-84549) |
| chr3 | 96727503 | -2.7989 | Rnf115 (-107), Polr3c (-63) |
| chr3  www.aging-us.com 3 AGING | 96919687 | 1.2607 | Gpr89 (-14342), Gja8 (+6332) |
| chr3 | 99141438 | -0.0605 | Wars2 (+371) |
| chr3 | 103809575 | -3.1059 | Dclre1b (-132), Ap4b1 (-52) |
| chr3 | 104220213 | -1.489 | Magi3 (-111) |
| chr3 | 107332143 | 0.2376 | Rbm15 (+1145), Slc16a4 (+40914) |
| chr3 | 107517398 | -0.1518 | Slc6a17 (+619) |
| chr3 | 107517805 | 0.0064 | Slc6a17 (+212) |
| chr3 | 108100140 | -1.0069 | Gnat2 (+7352), Gnai3 (+46005) |
| chr3 | 121507851 | -0.2904 | Slc44a3 (+24492), Cnn3 (+81311) |
| chr3 | 121545526 | -0.734 | Slc44a3 (-13183), A730020M07Rik (+97958) |
| chr3 | 133310529 | 4.6241 | Ppa2 (+420) |
| chr3 | 135593759 | 0.4888 | Nfkb1 (+97786) |
| chr3 | 142169526 | 3.7726 | NONE |
| chr3 | 148817552 | -0.2729 | NONE |
| chr3 | 153500769 | 0.2055 | NONE |
| chr4 | 28140436 | 0.1074 | NONE |
| chr4 | 33209344 | -2.4876 | Srsf12 (+354) |
| chr4 | 42924950 | 0.6803 | Dnajb5 (-25445), N28178 (+7700) |
| chr4 | 43382620 | -0.0526 | Rusc2 (-23814) |
| chr4 | 44090814 | 0.7814 | Gne (-6638), Rnf38 (+76793) |
| chr4 | 45342692 | -0.6659 | Dcaf10 (+592) |
| chr4 | 45531471 | -4.8567 | Shb (-1142) |
| chr4 | 55071885 | 0.7197 | NONE |
| chr4 | 57142836 | 2.8598 | Epb4.1l4b (+600) |
| chr4 | 63213975 | -0.3743 | Col27a1 (-1459) |
| chr4 | 83323961 | 0.2172 | Ttc39b (+293) |
| chr4 | 89294317 | 1.203 | Cdkn2a (+301) |
| chr4 | 89688406 | 0.0932 | Dmrta1 (+209) |
| chr4 | 91376687 | 0.0048 | Elavl2 (+23296) |
| chr4 | 96306742 | 0.4414 | Cyp2j7-ps (-70084), Cyp2j11 (+41919) |
| chr4 | 98383107 | -3.9869 | Tm2d1 (+198) |
| chr4 | 99929714 | -0.6041 | Pgm2 (+301) |
| chr4 | 101496665 | -0.7584 | Dnajc6 (-53928), Ak4 (+77377) |
| chr4 | 107113675 | -0.5356 | Tmem59 (-64723), Cdcp2 (+16785) |
| chr4 | 107435699 | 0.0317 | Glis1 (+981) |
| chr4 | 108848499 | -5.7843 | A730015C16Rik (+119), Kti12 (+643) |
| chr4 | 110970207 | 0.225 | NONE |
| chr4 | 116627157 | 0.3995 | Nasp (+783) |
| chr4 | 118216098 | -0.2433 | Kdm4a (-36056), Ptprf (+75306) |
| chr4 | 119170566 | -0.4584 | Zfp691 (+3289), Slc2a1 (+61856) |
| chr4 | 120731318 | 0.0324 | Kcnq4 (+15929), Cited4 (+64756) |
| chr4 | 124882509 | 1.2967 | Epha10 (+1611), Cdca8 (+54407) |
| chr4 | 124882524 | 1.1965 | Epha10 (+1626), Cdca8 (+54392) |
| chr4 | 124882529 | 0.6438 | Epha10 (+1631), Cdca8 (+54387) |
| chr4 | 124882557 | 0.1964 | Epha10 (+1659), Cdca8 (+54359) |
| chr4  www.aging-us.com 4 AGING | 128654947 | 0.0001 | Phc2 (+246) |
| chr4 | 128743517 | -0.7919 | A3galt2 (-15740), Phc2 (+88816) |
| chr4 | 129118461 | 0.0532 | Hpca (+3574), Tmem54 (+12914) |
| chr4 | 129440744 | -1.6999 | Zbtb8b (+73) |
| chr4 | 129960125 | -3.6198 | Bai2 (-25038), Spocd1 (+12405) |
| chr4 | 131748757 | 0.7609 | Ptpru (+89473) |
| chr4 | 132535763 | -2.6772 | Atpif1 (-2105), Dnajc8 (+214) |
| chr4 | 132768755 | -0.3314 | Rpa2 (+421) |
| chr4 | 133062635 | -0.1272 | Wasf2 (-67869), Ahdc1 (+51130) |
| chr4 | 133518983 | 0.7495 | 1810019J16Rik (-8) |
| chr4 | 133752083 | -0.0709 | Pigv (-79437), Arid1a (+1527) |
| chr4 | 133753125 | -2.1905 | Arid1a (+485) |
| chr4 | 134357339 | -1.8766 | Slc30a2 (+14134), Extl1 (+15218) |
| chr4 | 134671566 | -0.0475 | Man1c1 (+32723) |
| chr4 | 135398147 | -1.3894 | Ncmap (+73) |
| chr4 | 137796064 | 0.3173 | Alpl (+319) |
| chr4 | 137993353 | -0.5859 | Eif4g3 (-102) |
| chr4 | 140034539 | 0.2282 | Klhdc7a (-66514) |
| chr4 | 141475368 | -0.1827 | Zbtb17 (+30715), Spen (+63228) |
| chr4 | 142239113 | 3.2259 | Kazn (+287) |
| chr4 | 147971677 | 1.2534 | Nppb (-14110), 2510039O18Rik (+30783) |
| chr4 | 151145281 | -0.3245 | Gm13090 (+55712) |
| chr4 | 152274815 | 1.1634 | Gpr153 (+454) |
| chr4 | 153957446 | 0.1838 | A430005L14Rik (+210) |
| chr5 | 108367192 | -1.6633 | Gm10419 (-2665) |
| chr5 | 117318929 | -0.6517 | Vsig10 (-336), Gm10399 (+312) |
| chr5 | 118331517 | -1.0276 | 2410131K14Rik (+86291) |
| chr6 | 83109176 | -1.9186 | Mrpl53 (+82) |
| chr7 | 44496696 | 10.3943 | Emc10 (-184), Fam71e1 (+109) |
| chr7 | 44496714 | 4.3211 | Emc10 (-202), Fam71e1 (+127) |
| chr8 | 119446522 | -0.4419 | Necab2 (-196) |
| chr8 | 122376301 | -0.8344 | Zc3h18 (-307) |
| chr9 | 18292401 | -1.1283 | Chordc1 (+135) |
| chr9 | 21161579 | 0.8823 | Pde4a (-4134) |
| chr10 | 5806177 | -0.181 | Fbxo5 (-578) |
| chr10 | 11343499 | -0.0466 | Epm2a (+96) |
| chr10 | 12964274 | -1.0171 | Stx11 (-16) |
| chr10 | 13008952 | -2.5752 | Sf3b5 (+503) |
| chr10 | 18743528 | 2.5791 | D10Bwg1379e (+229) |
| chr10 | 18845199 | 0.3714 | Perp (+129) |
| chr10 | 21882255 | 1.3916 | Sgk1 (+72) |
| chr10 | 31915673 | 0.2824 | NONE |
| chr10 | 37136243 | 0.4727 | 5930403N24Rik (-3314), Marcks (+2676) |
| chr10 | 40302742 | -1.4518 | Amd1 (-555) |
| chr10 | 40883862 | -0.0596 | Cdc40 (-720), Wasf1 (+36) |
| chr10  www.aging-us.com 5 AGING | 42582269 | 0.3944 | Nr2e1 (+1362), Snx3 (+80239) |
| chr10 | 42698541 | 2.3321 | Sec63 (-62954), Ostm1 (+19626) |
| chr10 | 43021928 | 2.8693 | NONE |
| chr10 | 45485682 | 5.3281 | Hace1 (-92146), Lin28b (-15482) |
| chr10 | 45485688 | 0.0949 | Hace1 (-92140), Lin28b (-15488) |
| chr10 | 45485699 | 3.486 | Hace1 (-92129), Lin28b (-15499) |
| chr10 | 45485702 | 1.381 | Hace1 (-92126), Lin28b (-15502) |
| chr10 | 45485738 | 2.9357 | Hace1 (-92090), Lin28b (-15538) |
| chr10 | 45485740 | 0.1506 | Hace1 (-92088), Lin28b (-15540) |
| chr10 | 48536401 | -1.8102 | NONE |
| chr10 | 58813472 | 0.0658 | Sh3rf3 (+15) |
| chr10 | 60687035 | -0.0926 | Cdh23 (+9454) |
| chr10 | 61783640 | -0.5302 | H2afy2 (+506) |
| chr10 | 75926527 | 0.0055 | Smarcb1 (-4911) |
| chr10 | 75969946 | 3.628 | Zfp280b (-62707), Gm5134 (+15433) |
| chr10 | 77978634 | -0.2506 | 1810043G02Rik (+111) |
| chr10 | 77988732 | -0.0118 | 1810043G02Rik (+10209), Pfkl (+21074) |
| chr10 | 78592411 | 0.178 | Syde1 (-448) |
| chr10 | 80265016 | -3.8795 | Gamt (-4005), Dazap1 (-57) |
| chr10 | 80265056 | -0.876 | Gamt (-4045), Dazap1 (-17) |
| chr10 | 80292702 | 5.3708 | Rps15 (+250) |
| chr10 | 80347708 | -1.8055 | Adamtsl5 (+703) |
| chr10 | 80347711 | -0.0249 | Adamtsl5 (+700) |
| chr10 | 80395229 | -0.218 | Mex3d (-7579), Mbd3 (+4320) |
| chr10 | 81070184 | -2.6459 | Thop1 (+150) |
| chr10 | 81268194 | -0.5064 | Mrpl54 (-1261), Apba3 (+23) |
| chr10 | 83648356 | -1.4938 | Appl2 (+381) |
| chr10 | 84883024 | -0.6837 | Ric8b (-34591) |
| chr10 | 85184938 | -4.8334 | Cry1 (+115) |
| chr10 | 86022132 | -2.1453 | Fbxo7 (+161) |
| chr10 | 91872716 | 0.3618 | NONE |
| chr10 | 95254036 | -0.0105 | Cradd (+70060) |
| chr10 | 116581900 | -1.8536 | Cnot2 (-401) |
| chr10 | 116980687 | -1.3597 | Gm10271 (-8079), Best3 (-5626) |
| chr10 | 119453833 | -1.3904 | NONE |
| chr10 | 121150427 | -0.0649 | NONE |
| chr10 | 121607037 | -0.4645 | Tbk1 (-20244), Xpot (+19278) |
| chr10 | 122047655 | 6.2145 | Srgap1 (-341) |
| chr10 | 122047709 | 4.7728 | Srgap1 (-395) |
| chr10 | 122047716 | 2.1529 | Srgap1 (-402) |
| chr10 | 122047719 | 2.2 | Srgap1 (-405) |
| chr10 | 122047722 | 1.095 | Srgap1 (-408) |
| chr10 | 122047725 | 1.3517 | Srgap1 (-411) |
| chr10 | 122047735 | 1.8355 | Srgap1 (-421) |
| chr10 | 123265554 | -0.7416 | Fam19a2 (+1479) |
| chr10  www.aging-us.com 6 AGING | 128589619 | 0.7731 | Erbb3 (+32) |
| chr10 | 128910113 | -2.0667 | Cd63 (+1195), Rdh5 (+9183) |
| chr10 | 129817110 | 0.7567 | Olfr811 (-14588), Olfr812 (+25951) |
| chr11 | 4160437 | 3.293 | Ccdc157 (-147), Sf3a1 (+88) |
| chr11 | 19264825 | 0.3815 | NONE |
| chr11 | 21993365 | 0.5756 | Otx1 (+8249) |
| chr11 | 22003790 | 1.9751 | Otx1 (-2176) |
| chr11 | 30771718 | -0.2449 | Psme4 (-7) |
| chr11 | 32532863 | -7.0998 | Stk10 (-441) |
| chr11 | 36023996 | -0.3175 | Wwc1 (-43470) |
| chr11 | 43404722 | -0.3589 | Pttg1 (+21240) |
| chr11 | 45980348 | 1.1446 | Sox30 (+39) |
| chr11 | 45980366 | 1.6378 | Sox30 (+57) |
| chr11 | 45980375 | 5.2747 | Sox30 (+66) |
| chr11 | 45980409 | 1.1938 | Sox30 (+100) |
| chr11 | 51650662 | -0.627 | D930048N14Rik (-293), N4bp3 (+179) |
| chr11 | 52167199 | 1.0631 | Olfr1373 (-21672), Olfr1371 (+46787) |
| chr11 | 58937311 | -1.2423 | Rnf187 (+1604), Btnl10 (+19255) |
| chr11 | 59307150 | -7.7181 | Wnt9a (+221) |
| chr11 | 60205837 | -0.9685 | Srebf1 (+14766) |
| chr11 | 60210617 | -1.055 | Srebf1 (+9986) |
| chr11 | 61684505 | 1.6177 | Fam83g (+87) |
| chr11 | 68901666 | 3.8404 | Rpl26 (+32) |
| chr11 | 69903212 | -0.3261 | 2810408A11Rik (-2256), Neurl4 (+1322) |
| chr11 | 69935790 | 0.1751 | Ybx2 (-5) |
| chr11 | 69935810 | 6.49 | Ybx2 (+15) |
| chr11 | 72410820 | -0.3781 | Smtnl2 (+892) |
| chr11 | 72913128 | -0.8995 | Atp2a3 (-48040) |
| chr11 | 73041046 | 0.5027 | 1200014J11Rik (-6736), Camkk1 (+22004) |
| chr11 | 74771174 | 5.6064 | Mettl16 (+345) |
| chr11 | 75588406 | -0.6308 | Pitpna (+310) |
| chr11 | 77489322 | 3.2982 | Git1 (-4239), Ankrd13b (+355) |
| chr11 | 77500543 | -0.1726 | Git1 (+6982), Trp53i13 (+12826) |
| chr11 | 77508522 | -0.0126 | Trp53i13 (+4847), Git1 (+14961) |
| chr11 | 78159974 | -0.7922 | Traf4 (+5614), Fam222b (+44369) |
| chr11 | 78436161 | 1.3299 | Slc46a1 (-29535), Slc13a2 (-13945) |
| chr11 | 79711616 | 0.5679 | NONE |
| chr11 | 84068227 | -5.6241 | Dusp14 (+573) |
| chr11 | 93885266 | -1.4732 | Mbtd1 (-585), Utp18 (+499) |
| chr11 | 95299998 | -0.6317 | Kat7 (+10247), Tac4 (+38470) |
| chr11 | 95413854 | 0.4176 | Spop (-243) |
| chr11 | 96007418 | 3.7627 | Igf2bp1 (-1479) |
| chr11 | 96777603 | 0.0753 | Snx11 (-73) |
| chr11 | 97663598 | -3.7182 | Mllt6 (+185) |
| chr11 | 98294584 | -0.8304 | Neurod2 (+35063), Cdk12 (+91250) |
| chr11  www.aging-us.com 7 AGING | 101120008 | 0.4349 | Fam134c (-116), Tubg1 (+71) |
| chr11 | 101186660 | -1.2047 | Cntnap1 (+10620), Ezh1 (+39789) |
| chr11 | 102438402 | 0.8226 | Grn (+8088), Fam171a2 (+9279) |
| chr11 | 102606024 | 1.226 | Gm1564 (-59563), Fzd2 (+1594) |
| chr11 | 103101452 | -0.312 | Acbd4 (-235), Plcd3 (+205) |
| chr11 | 105943925 | -0.2948 | Cyb561 (+486) |
| chr11 | 107468861 | -1.4165 | Pitpnc1 (+1837) |
| chr11 | 109298113 | -3.3638 | Rgs9 (+15) |
| chr11 | 115250261 | -0.2034 | Grin2c (+16981), Tmem104 (+62775) |
| chr11 | 115258227 | 3.4743 | Grin2c (+9015), Tmem104 (+70741) |
| chr11 | 116004961 | 1.3575 | Galk1 (+7757), Itgb4 (+30237) |
| chr11 | 116110430 | -1.9925 | Trim47 (-185) |
| chr11 | 116844188 | -0.8262 | Jmjd6 (-740), Mettl23 (+911) |
| chr11 | 117849480 | 3.462 | Birc5 (+230) |
| chr11 | 118474914 | -0.1053 | Engase (-1914) |
| chr11 | 118477189 | 1.2761 | Engase (+361) |
| chr11 | 120201542 | -0.2723 | Slc38a10 (-50197), Bahcc1 (-31404) |
| chr11 | 120234059 | -0.1151 | Bahcc1 (+1113) |
| chr11 | 120720724 | -1.8405 | Rac3 (-745) |
| chr11 | 120807077 | -0.249 | Dus1l (-10683), Fasn (+17100) |
| chr12 | 5050136 | -4.3324 | NONE |
| chr12 | 17176122 | 1.1748 | Kcnf1 (+765) |
| chr12 | 21373341 | -3.2525 | Adam17 (+290) |
| chr12 | 22156588 | 0.2805 | NONE |
| chr12 | 25119448 | -0.7126 | Id2 (-23357) |
| chr12 | 35535020 | 2.8261 | Ahr (+17) |
| chr12 | 40037857 | -1.1044 | Arl4a (-429) |
| chr12 | 51348013 | -0.2958 | G2e3 (-274) |
| chr12 | 52006533 | -1.3933 | Dtd2 (-33) |
| chr12 | 54203309 | 3.0601 | Egln3 (+550) |
| chr12 | 55493341 | -1.6924 | Nfkbia (-695) |
| chr12 | 56519991 | 0.0817 | Nkx2-1 (+16916) |
| chr12 | 57538782 | 0.7634 | Foxa1 (+7338) |
| chr12 | 57538787 | 5.8339 | Foxa1 (+7333) |
| chr12 | 65225772 | 0.0585 | Wdr20b (+256) |
| chr12 | 73794195 | -0.3459 | NONE |
| chr12 | 76405241 | 0.5157 | Ppp1r36 (-12357), Hspa2 (+1066) |
| chr12 | 78861250 | -4.7011 | Eif2s1 (-568), Atp6v1d (+387) |
| chr12 | 79276390 | -1.7629 | Zfyve26 (+19891), Rdh12 (+67477) |
| chr12 | 80766691 | -0.1488 | 4933426M11Rik (-23840), Ccdc177 (-6151) |
| chr12 | 80946119 | -0.3167 | Srsf5 (+535) |
| chr12 | 81314965 | 1.5704 | Slc8a3 (+18214) |
| chr12 | 83520400 | 1.3552 | Dcaf4 (-65) |
| chr12 | 84571363 | 0.5389 | Vsx2 (+1602), Abcd4 (+46102) |
| chr12 | 84698673 | 0.2173 | Syndig1l (+133) |
| chr12  www.aging-us.com 8 AGING | 84698708 | 1.3688 | Syndig1l (+98) |
| chr12 | 84876877 | 3.8583 | Ltbp2 (-383) |
| chr12 | 85816167 | -0.2293 | 0610007P14Rik (+8354), Mfsd7c (+69629) |
| chr12 | 86983575 | 0.288 | Zdhhc22 (+5100), 2310044G17Rik (+36233) |
| chr12 | 87254240 | -0.9997 | Noxred1 (-20685), Vipas39 (+12045) |
| chr12 | 87300033 | 5.3259 | Ism2 (-329) |
| chr12 | 91590228 | -0.0962 | Gtf2a1 (-220) |
| chr12 | 91778564 | -0.8152 | Ston2 (+7871) |
| chr12 | 98901031 | 0.0464 | Eml5 (+452) |
| chr12 | 99098589 | -0.6499 | NONE |
| chr12 | 99196738 | -0.8363 | NONE |
| chr12 | 101028820 | -1.1889 | Ccdc88c (+162) |
| chr12 | 104474946 | 3.095 | Gsc (-1620), Gm10000 (+459) |
| chr12 | 105948980 | 0.0123 | Vrk1 (-61282) |
| chr12 | 108334520 | 2.1797 | Cyp46a1 (+144) |
| chr12 | 111713206 | -1.9326 | Apopt1 (-73), Bag5 (+50) |
| chr12 | 111789447 | -0.1165 | Xrcc3 (+24393), Klc1 (+30599) |
| chr12 | 116405520 | -1.1435 | Ncapg2 (+119) |
| chr12 | 116485911 | 4.8465 | Ptprn2 (+192) |
| chr12 | 117875704 | -0.7688 | Cdca7l (+32190) |
| chr12 | 118848986 | 0.6535 | Sp8 (+2658) |
| chr13 | 12861299 | -0.9193 | Prl2c3 (-61222) |
| chr13 | 21995466 | 0.2684 | Prss16 (+14274), Pom121l2 (+14286) |
| chr13 | 23309646 | 2.0166 | Zfp322a (+59561), Vmn1r223 (+60409) |
| chr13 | 23555565 | 1.5266 | Hist1h4f (-3918), Hist1h1d (+534) |
| chr13 | 31625884 | -0.4904 | Foxf2 (+69) |
| chr13 | 34994035 | -0.8163 | Eci2 (+108) |
| chr13 | 37826677 | -4.0402 | Rreb1 (+363) |
| chr13 | 40723104 | 4.3725 | Tfap2a (+10718), Gm9979 (+19100) |
| chr13 | 40723180 | 7.0868 | Tfap2a (+10642), Gm9979 (+19176) |
| chr13 | 40723198 | 4.0866 | Tfap2a (+10624), Gm9979 (+19194) |
| chr13 | 48967798 | 0.3392 | Fam120a (+218) |
| chr13 | 49194999 | -1.4585 | Ninj1 (+7515), 1110007C09Rik (+21026) |
| chr13 | 49379830 | -0.3239 | Ippk (-41480), Bicd2 (+38282) |
| chr13 | 53476064 | -2.015 | Msx2 (-2991) |
| chr13 | 54071980 | -3.1999 | Sfxn1 (+136) |
| chr13 | 55471967 | 0.9425 | Prr7 (+7701), Dbn1 (+16143) |
| chr13 | 62944729 | -1.3408 | 2010111I01Rik (-70204), Fbp1 (-56448) |
| chr13 | 64274375 | 1.5726 | Cdc14b (+597) |
| chr13 | 64274399 | 0.1129 | Cdc14b (+573) |
| chr13 | 64274685 | 3.6013 | Cdc14b (+287) |
| chr13 | 73603991 | -2.2895 | Clptm1l (-10) |
| chr13 | 73806327 | -0.1223 | Nkd2 (+41303), Slc12a7 (+42631) |
| chr13 | 73816185 | -0.182 | Nkd2 (+31445), Slc12a7 (+52489) |
| chr13 | 73937741 | -1.8018 | Brd9 (-96), Trip13 (+25) |
| chr13  www.aging-us.com 9 AGING | 75943555 | 3.043 | Rhobtb3 (+311) |
| chr13 | 80886143 | -5.9244 | Arrdc3 (+2734) |
| chr13 | 94285121 | -0.5838 | Scamp1 (+707) |
| chr13 | 97198240 | -0.2351 | Hexb (+116) |
| chr13 | 98355249 | 0.1636 | Foxd1 (+1005) |
| chr13 | 98366996 | 0.3747 | Foxd1 (+12752) |
| chr13 | 98534239 | -2.8587 | NONE |
| chr13 | 103920603 | 1.6312 | Erbb2ip (-90) |
| chr13 | 108316541 | -1.1211 | Depdc1b (+210) |
| chr13 | 117025760 | -0.2143 | Parp8 (-245) |
| chr14 | 7817971 | 0.9657 | Flnb (+15) |
| chr14 | 16574885 | -0.035 | Rarb (+586) |
| chr14 | 20661648 | -0.0898 | Myoz1 (-5109), Synpo2l (+6657) |
| chr14 | 26670135 | -1.0564 | Pde12 (-253) |
| chr14 | 30715394 | 2.2829 | Sfmbt1 (-220) |
| chr14 | 32038440 | -0.1781 | Oxnad1 (-46972), Galnt15 (+9452) |
| chr14 | 34549482 | -2.6284 | 9230112D13Rik (-26682), Ldb3 (+39198) |
| chr14 | 45388538 | -0.5542 | Gnpnat1 (+263) |
| chr14 | 48655725 | 0.7035 | Otx2 (+9520) |
| chr14 | 49066516 | -1.2928 | Ap5m1 (+22), Exoc5 (+136) |
| chr14 | 57889849 | 2.6383 | Zdhhc20 (+412) |
| chr14 | 60691600 | -0.7035 | C1qtnf9 (-76533), Spata13 (+56872) |
| chr14 | 62953044 | -2.3582 | Defb48 (+31465) |
| chr14 | 68123821 | 0.5269 | Nefm (+1024), Nefl (+39959) |
| chr14 | 68125711 | 2.2618 | Nefm (-866) |
| chr14 | 70079547 | 0.3499 | Bin3 (-20597), Egr3 (+2103) |
| chr14 | 70453118 | -0.0123 | Phyhip (-4357) |
| chr14 | 70599470 | -5.5231 | Fam160b2 (+364) |
| chr14 | 75131208 | 15.5827 | Lrrc63 (-328) |
| chr14 | 77036497 | 0.9218 | Ccdc122 (-274), Lacc1 (+143) |
| chr14 | 79183069 | -0.7507 | Zfp957 (+64226) |
| chr14 | 104466428 | 0.6316 | Pou4f1 (+1562) |
| chr14 | 104473125 | -0.1504 | Pou4f1 (-5135), Rnf219 (+49540) |
| chr14 | 115040601 | -1.0888 | Gpc5 (-52024) |
| chr14 | 118180510 | -1.8896 | Gpr180 (+43384), Sox21 (+56519) |
| chr14 | 119858380 | 3.0738 | NONE |
| chr14 | 121275623 | -1.7219 | NONE |
| chr14 | 122107411 | -0.4301 | Tm9sf2 (+374) |
| chr14 | 122450733 | -2.1188 | Zic5 (+14943) |
| chr14 | 122463668 | 0.183 | Zic5 (+2008) |
| chr14 | 122479059 | 0.1693 | Gm10837 (-11526), Zic2 (+3625) |
| chr14 | 122479076 | 0.622 | Gm10837 (-11509), Zic2 (+3642) |
| chr15 | 8262884 | 0.0471 | 2410089E03Rik (+93779) |
| chr15 | 26308775 | 0.5312 | March11 (-288) |
| chr15 | 27681414 | 6.3673 | Fam105a (+127) |
| chr15  www.aging-us.com 10 AGING | 30172936 | 0.0882 | Ctnnd2 (+344) |
| chr15 | 35155861 | -2.3963 | Stk3 (-56) |
| chr15 | 35296880 | 3.8018 | Osr2 (+783) |
| chr15 | 37234227 | 0.1991 | Grhl2 (+1192) |
| chr15 | 39197639 | 0.2606 | Rims2 (-723) |
| chr15 | 41788827 | -3.1516 | Abra (+80892) |
| chr15 | 60823769 | 1.2664 | Fam84b (+1310) |
| chr15 | 62038016 | -4.5901 | Myc (+52626) |
| chr15 | 73173517 | -0.9409 | Ago2 (+11322), Chrac1 (+83126) |
| chr15 | 75746470 | 2.8546 | Mafa (+1451), Rhpn1 (+42191) |
| chr15 | 76476953 | -0.1844 | Hsf1 (-491), Bop1 (+315) |
| chr15 | 76722828 | 1.5148 | Lrrc24 (-656) |
| chr15 | 76817911 | -0.5911 | Arhgap39 (+171) |
| chr15 | 78174204 | 3.9576 | Ift27 (-97) |
| chr15 | 78174214 | 4.2981 | Ift27 (-107) |
| chr15 | 78428757 | -1.0432 | Kctd17 (+130) |
| chr15 | 78913985 | 3.1472 | Pdxp (+67) |
| chr15 | 78914200 | 5.4856 | Pdxp (+282) |
| chr15 | 78914302 | 6.4314 | Pdxp (+384) |
| chr15 | 78914407 | 0.7485 | Pdxp (+489) |
| chr15 | 82279433 | -0.5934 | Wbp2nl (-19550), Sept3 (+4499) |
| chr15 | 84756644 | 0.5386 | Arhgap8 (+36593), Phf21b (+99404) |
| chr15 | 85206037 | 5.0552 | Fbln1 (+89) |
| chr15 | 89453736 | 0.0112 | Mapk8ip2 (-176) |
| chr15 | 94910196 | 0.1824 | NONE |
| chr15 | 98780511 | 2.6396 | Wnt10b (-2362) |
| chr15 | 99030446 | -0.6849 | Tuba1c (+556) |
| chr15 | 99471618 | -0.468 | Nckap5l (-13871), Bcdin3d (+3111) |
| chr15 | 101929872 | 0.2939 | Krt4 (-5138), Krt79 (+10451) |
| chr15 | 101929878 | 0.7396 | Krt4 (-5144), Krt79 (+10445) |
| chr15 | 102279425 | -1.4774 | Mfsd5 (-30) |
| chr15 | 102975989 | 0.3893 | Hoxc9 (-1042) |
| chr16 | 3847098 | -0.0212 | Zfp174 (-169) |
| chr16 | 3872336 | 0.0892 | Zfp597 (+59) |
| chr16 | 3872650 | 5.6789 | Zfp597 (-255) |
| chr16 | 10313919 | 0.4157 | Emp2 (+48) |
| chr16 | 10502161 | 2.0708 | Ciita (+13984), Dexi (+40892) |
| chr16 | 11253937 | -5.4943 | Gspt1 (+271) |
| chr16 | 13256105 | 0.5157 | Mkl2 (-375) |
| chr16 | 14361658 | 0.2065 | Abcc1 (+99) |
| chr16 | 17798418 | 0.0796 | Scarf2 (+1137), Car15 (+39945) |
| chr16 | 17891609 | -1.3337 | Tssk1 (-2593), Gm20518 (+61), Dgcr2 (+83) |
| chr16 | 19133634 | 0.761 | Iglc2 (+66716) |
| chr16 | 20651732 | -0.3575 | Psmd2 (+81) |
| chr16 | 23107824 | -2.0557 | Eif4a2 (+381) |
| chr16  www.aging-us.com 11 AGING | 30587877 | -3.8351 | Lsg1 (-286) |
| chr16 | 30600158 | -0.0539 | Fam43a (+436) |
| chr16 | 33250171 | -1.6686 | Snx4 (-1284) |
| chr16 | 33830489 | -0.1347 | Itgb5 (+813) |
| chr16 | 35022825 | -1.8644 | Ptplb (+405) |
| chr16 | 87354566 | 0.0022 | N6amt1 (+382) |
| chr16 | 95987673 | -0.8504 | Psmg1 (+3286) |
| chr17 | 3084720 | -2.0387 | Scaf8 (-30251) |
| chr17 | 23673823 | 1.6617 | Thoc6 (+18), Hcfc1r1 (+216) |
| chr17 | 24606227 | -0.0898 | Tsc2 (+26401), Pkd1 (+56278) |
| chr17 | 26415515 | -0.4691 | Neurl1b (+551) |
| chr17 | 26849776 | 0.4762 | Kifc5b (-67314), Nkx2-5 (-8212) |
| chr17 | 26850736 | 0.4543 | Kifc5b (-66354), Nkx2-5 (-9172) |
| chr17 | 28177432 | -0.0192 | Zfp523 (+15) |
| chr17 | 28622722 | 1.0282 | Srpk1 (-202) |
| chr17 | 28909688 | 0.8915 | 4930539E08Rik (+5635), Pnpla1 (+51278) |
| chr17 | 34647456 | -5.6272 | Atf6b (+277) |
| chr17 | 34850397 | 1.5783 | Skiv2l (-188), Nelfe (+7) |
| chr17 | 35821867 | -0.0061 | Flot1 (-1362), Ier3 (+184) |
| chr17 | 35894940 | 0.4656 | 2310061I04Rik (+2490), Dhx16 (+15122) |
| chr17 | 45576099 | -0.0418 | Hsp90ab1 (-2829) |
| chr17 | 45664987 | -1.3998 | Capn11 (-5663), Tmem63b (+21271) |
| chr17 | 46674224 | 2.4034 | Rrp36 (+30) |
| chr17 | 46687337 | -0.0066 | Mea1 (+6201), Ppp2r5d (+17664) |
| chr17 | 50292979 | 0.5307 | Dazl (+619) |
| chr17 | 50292987 | 0.9001 | Dazl (+611) |
| chr17 | 53689264 | 2.2493 | Sgol1 (+68) |
| chr17 | 55878844 | -2.0315 | Zfp119a (+17) |
| chr17 | 55970486 | -2.0348 | Shd (+20) |
| chr17 | 56626659 | 11.7816 | Catsperd (-1483), Lonp1 (+243) |
| chr17 | 56935828 | 6.7322 | Mllt1 (-441) |
| chr17 | 56935839 | 3.116 | Mllt1 (-452) |
| chr17 | 57081607 | -0.336 | Dennd1c (-3098) |
| chr17 | 69969337 | 1.5705 | NONE |
| chr17 | 69969391 | 2.4959 | NONE |
| chr17 | 73710569 | -5.0901 | Galnt14 (-119) |
| chr17 | 80373376 | 3.7484 | Arhgef33 (+65970) |
| chr17 | 84185908 | -2.1685 | Zfp36l2 (+2038) |
| chr17 | 84466096 | -9.4537 | Thada (+99) |
| chr17 | 86970229 | 0.7504 | Rhoq (+7148), Pigf (+55176) |
| chr17 | 87258111 | -0.441 | Mcfd2 (+7792) |
| chr17 | 87975219 | -5.8666 | Msh6 (+170) |
| chr18 | 3336813 | -0.5116 | Crem (+865) |
| chr18 | 6241331 | -2.2163 | Kif5b (+191) |
| chr18 | 7003400 | -0.0426 | Mkx (+1313) |
| chr18  www.aging-us.com 12 AGING | 11048438 | 0.1691 | Gata6 (-4071) |
| chr18 | 11839608 | 0.806 | Cables1 (+389) |
| chr18 | 34355772 | -2.7749 | Reep5 (+17642), Srp19 (+24628) |
| chr18 | 34793123 | -0.3263 | Reep2 (-47465), Kdm3b (+16116) |
| chr18 | 36018245 | 1.085 | Psd2 (+53416) |
| chr18 | 36196614 | -0.6189 | Nrg2 (+765) |
| chr18 | 36320255 | -0.3168 | Cystm1 (-28518), Pura (+39159) |
| chr18 | 36320621 | 1.2509 | Cystm1 (-28152), Pura (+39525) |
| chr18 | 37807726 | 0.2093 | Pcdhga11 (-11819) |
| chr18 | 38841353 | 0.0017 | Fgf1 (+77417) |
| chr18 | 39489490 | -2.8241 | Nr3c1 (+1183) |
| chr18 | 42511094 | -1.2144 | Tcerg1 (-415) |
| chr18 | 49531789 | 0.372 | NONE |
| chr18 | 66061561 | -1.7962 | Lman1 (-58925) |
| chr18 | 76861061 | -0.9896 | Ier3ip1 (-68955), Skor2 (+4657) |
| chr18 | 78858827 | 0.3438 | NONE |
| chr18 | 83051667 | -0.1768 | NONE |
| chr18 | 83051760 | -2.208 | NONE |
| chr18 | 84685444 | 0.5454 | Cndp2 (-3449) |
| chr19 | 4214299 | -1.5136 | Clcf1 (+62) |
| chr19 | 4397285 | 0.7259 | Kdm2a (-209) |
| chr19 | 4613765 | -0.563 | Lrfn4 (+1736) |
| chr19 | 4756363 | -8.9538 | Rbm4b (-207) |
| chr19 | 5742232 | -0.6146 | Ltbp3 (+1329), Scyl1 (+29168) |
| chr19 | 6141058 | -2.0543 | Arl2 (+347) |
| chr19 | 8941842 | -4.1411 | Mta2 (-32) |
| chr19 | 16436756 | 2.9055 | Gna14 (+1090) |
| chr19 | 18713622 | -1.3906 | D030056L22Rik (+387) |
| chr19 | 27429839 | -0.3173 | D19Bwg1357e (-20) |
| chr19 | 29648305 | -2.5417 | Ermp1 (+109) |
| chr19 | 30030662 | 2.733 | Uhrf2 (+150) |
| chr19 | 30175122 | 2.0507 | Gldc (+295) |
| chr19 | 31762866 | 2.0969 | Prkg1 (-98497) |
| chr19 | 34746690 | -0.3828 | Slc16a12 (+598) |
| chr19 | 36057657 | -0.1948 | Htr7 (-318) |
| chr19 | 37018643 | 0.0666 | Btaf1 (+92565) |
| chr19 | 37435318 | 1.9621 | Hhex (+2432) |
| chr19 | 40895172 | 3.5706 | Zfp518a (+468) |
| chr19 | 42518732 | 3.4777 | R3hcc1l (-26) |
| chr19 | 44750466 | 2.2784 | Pax2 (-5582) |
| chr19 | 47731600 | -2.07 | Sfr1 (-155) |
| chr19 | 53600187 | -4.0217 | Smc3 (-210) |
| chr19 | 53600843 | 2.7944 | Smc3 (+446) |
| chr19 | 56874114 | -0.5281 | Vwa2 (-134) |
| chr19 | 56906573 | -0.0905 | Vwa2 (+32325) |
| chr19  www.aging-us.com 13 AGING | 59459911 | 0.5128 | Emx2 (+1540) |
| chr19 | 61226356 | 0.3507 | Csf2ra (+2061), Gm6020 (+42467) |
| chr19 | 61226587 | 0.6196 | Csf2ra (+1830), Gm6020 (+42698) |
| chr19 | 61226635 | 0.4102 | Csf2ra (+1782), Gm6020 (+42746) |
| chr19 | 61226647 | 3.9526 | Csf2ra (+1770), Gm6020 (+42758) |
| chr19 | 61226650 | 3.356 | Csf2ra (+1767), Gm6020 (+42761) |
| chr19 | 61226668 | 2.1127 | Csf2ra (+1749), Gm6020 (+42779) |

www.aging-us.com 14 AGING
