# Supplementary material for: A multi-tissue full lifespan epigenetic clock for mice
Source: Aging (Albany NY). 2018 Oct 21;10(10):2832–54. doi: 10.18632/aging.101590 (PMC6224226; doi:10.18632/aging.101590)
Supplement: Supplementary Table 2 [file aging-10-101590-s003.docx]

**Supplementary Table 2.** CpGs utilized in the elastic net age clock derived from evolutionarily conserved CpGs for which methylation data was available. Listed are the genomic coordinates, the linear coefficients of the model, and the distances to the transcription site (TSS) of the nearest genes.

| **Chromosome** | **Coordinate** | **Coefficient** | **Proximal Genes (distance to TSS)** |
| --- | --- | --- | --- |
| intercept |  | 13.6378 |  |
| chr1 | 23922136 | -9.7072 | Smap1 (+180) |
| chr1 | 25829517 | 6.3764 | Gm9884 (-1139), Bai3 (+189) |
| chr1 | 74855811 | 1.0576 | Cdk5r2 (+783) |
| chr1 | 78196764 | 2.143 | Pax3 (+73) |
| chr1 | 1.21E+08 | 1.9216 | En1 (-2232) |
| chr1 | 1.21E+08 | 0.5832 | En1 (-103) |
| chr1 | 1.21E+08 | 19.7061 | En1 (-99) |
| chr1 | 1.33E+08 | -0.0861 | Sox13 (-4498) |
| chr1 | 1.88E+08 | 0.4047 | Esrrg (-54) |
| chr1 | 1.9E+08 | 7.9595 | Prox1 (+10) |
| chr1 | 1.92E+08 | -5.3734 | Slc30a1 (-38), 1700034H15Rik (+798) |
| chr2 | 6872384 | -5.2553 | Celf2 (+12611) |
| chr2 | 9896052 | 4.4571 | 9230102O04Rik (-12060) |
| chr2 | 13010915 | 4.6989 | C1ql3 (+831) |
| chr2 | 25292431 | -2.3374 | Lrrc26 (+2517), Grin1 (+26688) |
| chr2 | 30474002 | -2.1123 | Ier5l (+196) |
| chr2 | 31635948 | 0.3936 | Prdm12 (-4088) |
| chr2 | 33639563 | -9.9954 | Lmx1b (+947) |
| chr2 | 38348059 | 15.2712 | Lhx2 (-2699) |
| chr2 | 55437515 | 0.2038 | Kcnj3 (+1546) |
| chr2 | 71546351 | 4.4231 | Dlx2 (+402) |
| chr2 | 74135851 | 0.4665 | Lnp (+443096) |
| chr2 | 74682276 | 5.5981 | Hoxd10 (-8813), Hoxd11 (+278), Hoxd13 (+13967) |
| chr2 | 74727085 | 0.1012 | Hoxd3 (+358), Hoxd13 (+58776) |
| chr2 | 74734745 | 1.6732 | Hoxd1 (-28234), Hoxd3 (+8018), Hoxd13 (+66436) |
| chr2 | 74753208 | 2.2466 | Hoxd1 (-9771), Hoxd3 (+26481), Hoxd13 (+84899) |
| chr2 | 82053835 | 1.019 | Zfp804a (+614) |
| chr2 | 92055426 | 10.0113 | Creb3l1 (-30925) |
| chr2 | 94241431 | -3.8299 | Hsd17b12 (-83468) |
| chr2 | 1.04E+08 | -5.1725 | Abtb2 (+489) |
| chr2 | 1.06E+08 | 0.5472 | Pax6 (-699) |
| chr2 | 1.06E+08 | -1.0798 | Pax6 (+6140) |
| chr2 | 1.06E+08 | 2.6148 | Pax6 (+6147) |
| chr2 | 1.16E+08 | 1.2518 | NONE |
| chr2 | 1.19E+08 | -6.3255 | Ino80 (+73) |
| chr2 | 1.26E+08 | -1.3629 | Shc4 (+103) |
| chr2 | 1.29E+08 | -2.3333 | Gm10762 (-61), Zc3h6 (+384) |
| chr2 | 1.3E+08 | 3.1642 | Cpxm1 (+32123), Ebf4 (+69512) |
| chr2  www.aging-us.com 1 AGING | 1.35E+08 | -0.6039 | Plcb1 (+419) |
| chr2 | 1.37E+08 | 8.1965 | Snap25 (-3) |
| chr2 | 1.37E+08 | 6.4416 | Snap25 (+47) |
| chr2 | 1.47E+08 | 0.5332 | Nkx2-2 (-1845), 6430503K07Rik (+823) |
| chr2 | 1.47E+08 | -1.4554 | Nkx2-2 (-1885), 6430503K07Rik (+863) |
| chr2 | 1.47E+08 | 0.0302 | Nkx2-2 (-1899), 6430503K07Rik (+877) |
| chr2 | 1.47E+08 | 0.8454 | 6430503K07Rik (+7821) |
| chr2 | 1.48E+08 | 3.1988 | Foxa2 (+2624) |
| chr2 | 1.53E+08 | 0.4734 | 4930404H24Rik (-94863), Asxl1 (+52082) |
| chr2 | 1.56E+08 | -0.5751 | Ergic3 (+3144), Fer1l4 (+41758) |
| chr2 | 1.6E+08 | 7.0634 | Gm826 (-38371), Mafb (+1200) |
| chr2 | 1.65E+08 | 0.7629 | Slc12a5 (+274) |
| chr3 | 30996041 | 17.8674 | Prkci (+295) |
| chr3 | 45381862 | 5.28 | Pcdh10 (+3465) |
| chr3 | 52104065 | 8.7114 | Maml3 (+825) |
| chr3 | 73048527 | 0.4012 | Sis (-80665), Slitrk3 (+8415) |
| chr3 | 83766831 | 3.4908 | Sfrp2 (+511) |
| chr3 | 88140721 | -1.0008 | Mef2d (-1650) |
| chr3 | 88168282 | 1.0602 | Mef2d (+25911), Rhbg (+86426) |
| chr3 | 90292827 | 0.796 | Gatad2b (-48826), Dennd4b (+26314) |
| chr3 | 90292876 | -3.1864 | Gatad2b (-48777), Dennd4b (+26363) |
| chr3 | 1.03E+08 | -1.1359 | Csde1 (+93) |
| chr3 | 1.03E+08 | -0.3024 | Csde1 (+110) |
| chr3 | 1.17E+08 | -8.5877 | D3Bwg0562e (+846) |
| chr3 | 1.22E+08 | -7.2073 | Dnttip2 (+18541) |
| chr3 | 1.22E+08 | -0.2841 | Dnttip2 (+18557) |
| chr3 | 1.22E+08 | -5.2626 | Dnttip2 (+18608) |
| chr3 | 1.29E+08 | 1.2474 | Pitx2 (+5151) |
| chr3 | 1.29E+08 | 3.2842 | Pitx2 (+5157) |
| chr3 | 1.29E+08 | 0.5572 | Pitx2 (+5163) |
| chr3 | 1.34E+08 | -6.7666 | Tet2 (+42) |
| chr3 | 1.38E+08 | -0.0688 | Metap1 (+120) |
| chr3 | 1.57E+08 | 9.8586 | Negr1 (+433) |
| chr3 | 1.57E+08 | 1.3334 | Negr1 (+451) |
| chr4 | 5799580 | 0.4053 | NONE |
| chr4 | 13784979 | -4.9343 | Runx1t1 (+33683) |
| chr4 | 21931713 | 0.5785 | Faxc (+383) |
| chr4 | 34882990 | -6.2963 | Zfp292 (-31) |
| chr4 | 49845025 | 0.747 | Grin3a (+523) |
| chr4 | 59626459 | 7.6667 | E130308A19Rik (+217) |
| chr4 | 65124588 | 5.8708 | Pappa (+415) |
| chr4 | 88031563 | 26.4114 | Mllt3 (+1800) |
| chr4 | 91375206 | -0.9707 | Elavl2 (+24777) |
| chr4 | 91381398 | 2.38 | Elavl2 (+18585) |
| chr4 | 1.07E+08 | -13.5263 | Ssbp3 (-83539), Acot11 (-28085) |
| chr4  www.aging-us.com 2 AGING | 1.1E+08 | 2.6039 | Cdkn2c (+531) |
| chr4 | 1.21E+08 | -4.5412 | Kcnq4 (+15958), Cited4 (+64727) |
| chr4 | 1.25E+08 | -1.0527 | Pou3f1 (+14794), Utp11l (+21160) |
| chr4 | 1.25E+08 | 1.5543 | Pou3f1 (+14918), Utp11l (+21036) |
| chr4 | 1.27E+08 | -1.2711 | Zmym4 (+39) |
| chr4 | 1.29E+08 | -1.3318 | A3galt2 (-15750), Phc2 (+88806) |
| chr4 | 1.29E+08 | -7.6282 | A3galt2 (-15740), Phc2 (+88816) |
| chr4 | 1.33E+08 | 0.384 | Fam46b (+6550), Trnp1 (+11868) |
| chr4 | 1.36E+08 | -1.5849 | Id3 (-2906) |
| chr4 | 1.36E+08 | 5.0007 | Hnrnpr (+45) |
| chr4 | 1.38E+08 | 0.2928 | Eif4g3 (+22) |
| chr4 | 1.38E+08 | 0.8479 | Fam43b (+628), AB041806 (+632) |
| chr4 | 1.39E+08 | 2.5725 | Ubr4 (+199) |
| chr4 | 1.4E+08 | 4.586 | Pax7 (+1890) |
| chr4 | 1.49E+08 | -0.094 | Gm572 (+33823) |
| chr4 | 1.49E+08 | 3.3604 | Casz1 (+1527) |
| chr4 | 1.49E+08 | -3.2782 | NONE |
| chr6 | 39873175 | -16.189 | Mrps33 (-62188) |
| chr6 | 72235506 | -9.4424 | Atoh8 (+70) |
| chr6 | 77243836 | -2.8226 | Lrrtm1 (+1140) |
| chr6 | 86525026 | 11.5603 | Pcbp1 (+1144), C87436 (+86653) |
| chr8 | 46294681 | -6.5016 | Helt (-11) |
| chr8 | 57328652 | 0.6829 | Hand2 (+7670) |
| chr8 | 57328658 | 3.0487 | Hand2 (+7676) |
| chr10 | 21991716 | -0.5351 | 4930444G20Rik (+76362) |
| chr10 | 29535953 | -3.1687 | Rspo3 (-87) |
| chr10 | 37138511 | 5.2529 | 5930403N24Rik (-1046), Marcks (+408) |
| chr10 | 42591774 | -4.8622 | Ostm1 (-87141), Nr2e1 (-8143) |
| chr10 | 59616647 | -3.5582 | Mcu (+44), Gm10322 (+585) |
| chr10 | 63023319 | 0.0035 | Pbld2 (-1192), Hnrnph3 (+574) |
| chr10 | 1.23E+08 | -5.9584 | Mon2 (+90882) |
| chr10 | 1.23E+08 | 16.5319 | Mon2 (+90811) |
| chr11 | 23255924 | 10.1184 | Xpo1 (-153) |
| chr11 | 23255943 | -2.6422 | Xpo1 (-134) |
| chr11 | 23255953 | -5.4485 | Xpo1 (-124) |
| chr11 | 23256016 | 13.7585 | Xpo1 (-61) |
| chr11 | 31870925 | 8.7115 | Cpeb4 (-1285) |
| chr11 | 33203551 | 6.3264 | Tlx3 (+36) |
| chr11 | 33203575 | 0.8599 | Tlx3 (+12) |
| chr11 | 33203648 | 10.4231 | Tlx3 (-61) |
| chr11 | 42182462 | 0.572 | Gabra1 (+467) |
| chr11 | 47379459 | -1.8186 | Sgcd (+62) |
| chr11 | 58307712 | -1.6692 | Zfp692 (+644) |
| chr11 | 58307734 | -2.5741 | Zfp692 (+666) |
| chr11 | 60417754 | -6.6415 | Atpaf2 (-704), Gid4 (+493) |
| chr11  www.aging-us.com 3 AGING | 69345523 | -2.7025 | Kcnab3 (+19266), Chd3 (+23882) |
| chr11 | 69345527 | -1.329 | Kcnab3 (+19270), Chd3 (+23878) |
| chr11 | 69361190 | -0.3672 | Chd3 (+8215), Kcnab3 (+34933) |
| chr11 | 69406378 | -2.3752 | Tmem88 (-8145), Kdm6b (+7296) |
| chr11 | 69406413 | -1.8075 | Tmem88 (-8180), Kdm6b (+7261) |
| chr11 | 74831407 | 11.9008 | Mnt (+488) |
| chr11 | 78154447 | -2.068 | Traf4 (+11141), Fam222b (+38842) |
| chr11 | 78154490 | 0.7741 | Traf4 (+11098), Fam222b (+38885) |
| chr11 | 96004885 | 1.5327 | B4galnt2 (-89995), Igf2bp1 (+1054) |
| chr11 | 96271184 | -2.1275 | Hoxb9 (-272) |
| chr11 | 96286697 | -2.7818 | Hoxb7 (+75) |
| chr11 | 98329605 | 2.7619 | Neurod2 (+42) |
| chr11 | 98329610 | 4.568 | Neurod2 (+37) |
| chr11 | 1.04E+08 | 7.3354 | Wnt3 (+17) |
| chr11 | 1.04E+08 | -1.2069 | Wnt3 (+87) |
| chr11 | 1.04E+08 | -1.2732 | Mapt (+86609) |
| chr11 | 1.16E+08 | -11.0986 | H3f3b (-2480), Unk (+120) |
| chr11 | 1.18E+08 | 2.2248 | Tha1 (-94664), Socs3 (+1063) |
| chr11 | 1.18E+08 | 0.1525 | Socs3 (+939) |
| chr11 | 1.19E+08 | 0.895 | Cbx8 (-1909) |
| chr12 | 3960115 | 0.6027 | Pomc (+5165), Efr3b (+78799) |
| chr12 | 12725223 | -1.7854 | NONE |
| chr12 | 33147856 | -0.6481 | Cdhr3 (-54982) |
| chr12 | 56524961 | -0.7087 | Nkx2-1 (+11946) |
| chr12 | 59219444 | -1.5996 | Fbxo33 (+280) |
| chr12 | 69373153 | 2.1344 | Gm9887 (-696) |
| chr12 | 73046733 | 0.4858 | Six1 (+445) |
| chr12 | 75596226 | 5.2605 | Ppp2r5e (-27) |
| chr12 | 76405316 | 7.0478 | Ppp1r36 (-12282), Hspa2 (+1141) |
| chr12 | 80170742 | -0.2967 | Zfp36l1 (-57730), Actn1 (+89628) |
| chr12 | 84569707 | 0.3779 | Vsx2 (-54) |
| chr12 | 99393039 | -10.5977 | Foxn3 (+32) |
| chr12 | 1.03E+08 | 4.3428 | Unc79 (+442) |
| chr12 | 1.06E+08 | -5.1526 | Gskip (-650), Atg2b (+539) |
| chr12 | 1.19E+08 | 0.9613 | Sp8 (-2332) |
| chr13 | 29986038 | -1.9395 | E2f3 (-371) |
| chr13 | 31559516 | 0.126 | A530084C06Rik (-184) |
| chr13 | 36734248 | 1.6302 | Nrn1 (+228) |
| chr13 | 55549159 | 2.3025 | Ddx41 (-12502), Fam193b (+21960) |
| chr13 | 55750471 | 0.972 | Catsper3 (-34096), Pcbd2 (+23104) |
| chr13 | 91461156 | 5.0197 | Ssbp2 (+107) |
| chr13 | 91461177 | 0.8408 | Ssbp2 (+128) |
| chr13 | 91461224 | 10.553 | Ssbp2 (+175) |
| chr13 | 94706910 | 4.5807 | Tbca (-82032) |
| chr13 | 94869342 | -4.7451 | Otp (-6284), Tbca (+80400) |
| chr13  www.aging-us.com 4 AGING | 94872594 | -1.6782 | Otp (-3032) |
| chr13 | 1.05E+08 | 6.0906 | Fam159b (+320) |
| chr13 | 1.14E+08 | -26.4447 | Snx18 (+285) |
| chr14 | 11553574 | 6.3769 | Ptprg (+43) |
| chr14 | 12345742 | 1.4455 | Fezf2 (+122) |
| chr14 | 12345745 | 0.2835 | Fezf2 (+119) |
| chr14 | 16574885 | -5.7127 | Rarb (+586) |
| chr14 | 16574952 | -8.8156 | Rarb (+519) |
| chr14 | 20741446 | 1.0967 | Ndst2 (-11120), Camk2g (+52641) |
| chr14 | 21027184 | 3.979 | Ap3m1 (+25265), Vcl (+97752) |
| chr14 | 21994520 | -1.4091 | Zfp503 (-4920) |
| chr14 | 24003777 | 1.3882 | NONE |
| chr14 | 45219745 | -4.8443 | Txndc16 (-352), Gpr137c (-247) |
| chr14 | 45329870 | -6.4414 | Psmc6 (+47) |
| chr14 | 45657545 | -1.4958 | Ddhd1 (+552) |
| chr14 | 46384349 | -2.0882 | Gm15217 (+4883), Bmp4 (+6319) |
| chr14 | 55114974 | -3.0178 | Ap1g2 (-8382), Jph4 (+1960) |
| chr14 | 63606670 | -2.1192 | Xkr6 (+168) |
| chr14 | 65235274 | 1.3251 | Fzd3 (+27188) |
| chr14 | 70079611 | 2.1466 | Bin3 (-20533), Egr3 (+2167) |
| chr14 | 93890725 | 9.8227 | Pcdh9 (-1994) |
| chr14 | 1.04E+08 | 3.4332 | Pou4f1 (-246) |
| chr14 | 1.17E+08 | 10.9055 | Gpc6 (+571) |
| chr14 | 1.22E+08 | 5.4603 | Zic5 (+11753) |
| chr14 | 1.22E+08 | 0.8551 | Gm10837 (-14055), Zic2 (+1096) |
| chr15 | 6386680 | -0.8991 | Dab2 (+83) |
| chr15 | 12170825 | -1.0013 | Mtmr12 (-34202), Zfr (+52995) |
| chr15 | 27466996 | 4.3647 | Ank (+320) |
| chr15 | 38078124 | -3.2307 | Ubr5 (+728) |
| chr15 | 79028764 | -2.9638 | Gcat (-2109), H1f0 (+553) |
| chr15 | 81585368 | -7.5177 | Ep300 (-845) |
| chr15 | 1.03E+08 | -0.3939 | Atf7 (-64643), Atf7 (+31753) |
| chr15 | 1.03E+08 | 0.5491 | Hoxc10 (+88) |
| chr15 | 1.03E+08 | 1.5484 | Hoxc5 (-4122), Hoxc6 (+214) |
| chr15 | 1.03E+08 | -0.7458 | Hoxc4 (+399) |
| chr15 | 1.03E+08 | 0.4239 | Hoxc4 (+456) |
| chr16 | 11984855 | 13.7056 | Shisa9 (+275) |
| chr16 | 20531469 | 2.5442 | Ap2m1 (-4010) |
| chr16 | 23987158 | -5.1278 | Rtp2 (-56372), Bcl6 (+1693) |
| chr16 | 24990532 | -0.1667 | NONE |
| chr16 | 28446159 | 1.1202 | Fgf12 (-933) |
| chr16 | 28929569 | -3.4568 | Mb21d2 (+128) |
| chr16 | 65815643 | 6.736 | Vgll3 (+11) |
| chr16 | 76373016 | 6.3122 | Nrip1 (+810) |
| chr17 | 5493464 | 20.6053 | Zdhhc14 (+865) |
| chr17  www.aging-us.com 5 AGING | 29928992 | 1.645 | Zfand3 (-76094), Mdga1 (-41111) |
| chr17 | 31564837 | -1.8486 | Pknox1 (+37) |
| chr17 | 44608121 | 2.8279 | NONE |
| chr17 | 46254520 | -3.0618 | Lrrc73 (+356) |
| chr17 | 46555364 | 0.1363 | Srf (+797) |
| chr17 | 46555387 | 7.3955 | Srf (+774) |
| chr17 | 46890373 | -4.6947 | Tbcc (-247), A330017A19Rik (+31) |
| chr17 | 47872575 | -3.335 | Mdfi (-37885), Foxp4 (+51859) |
| chr17 | 47877553 | 1.5825 | Mdfi (-42863), Foxp4 (+46881) |
| chr17 | 51536957 | 0.185 | NONE |
| chr17 | 56830934 | 1.7874 | Rfx2 (+73) |
| chr17 | 86168923 | -0.4747 | Prkce (+1139) |
| chr17 | 87746301 | 1.2169 | Kcnk12 (+51692), Msh2 (+73972) |
| chr18 | 3508216 | 4.4733 | Bambi (+260) |
| chr18 | 3508260 | 1.4028 | Bambi (+304) |
| chr18 | 6490579 | 3.5413 | Epc1 (+276) |
| chr18 | 25486855 | 4.8517 | NONE |
| chr18 | 25486866 | 1.854 | NONE |
| chr18 | 25753474 | 1.0865 | Celf4 (+508) |
| chr18 | 36196601 | 4.6533 | Nrg2 (+778) |
| chr18 | 45559398 | 1.8216 | NONE |
| chr18 | 60925553 | 1.1028 | Camk2a (-64) |
| chr18 | 64340023 | 5.9742 | Onecut2 (-340) |
| chr18 | 64341586 | 0.0394 | Onecut2 (+1223) |
| chr18 | 76379026 | 1.8835 | NONE |
| chr18 | 76533533 | 0.1248 | NONE |
| chr19 | 7268287 | -10.1823 | Rcor2 (-1476) |
| chr19 | 11966253 | -8.0379 | Osbp (+410) |
| chr19 | 11966308 | -2.8594 | Osbp (+465) |
| chr19 | 17199182 | -2.047 | NONE |
| chr19 | 23135911 | -3.7169 | Klf9 (-5314) |
| chr19 | 23141943 | -1.1511 | Klf9 (+718) |
| chr19 | 32159671 | -7.8069 | Asah2 (-98203) |
| chr19 | 32757670 | -13.1933 | Pten (+174) |
| chr19 | 44672172 | 1.0064 | Pax2 (-83876) |
| chr19 | 44672177 | 2.6729 | Pax2 (-83871) |
| chr19 | 44674870 | 0.1725 | Pax2 (-81178) |
| chr19 | 44674906 | 1.3817 | Pax2 (-81142) |
| chr19 | 44760882 | 2.7121 | Pax2 (+4834) |
| chr19 | 44760900 | 1.0906 | Pax2 (+4852) |
| chr19 | 44808947 | 1.9232 | Pax2 (+52899) |
| chr19 | 45225625 | -3.7558 | Lbx1 (+10185), Tlx1 (+76390) |
| chr19 | 45229430 | 1.6458 | Lbx1 (+6380), Tlx1 (+80195) |
| chr19 | 45231293 | 0.8332 | Lbx1 (+4517), Tlx1 (+82058) |
| chr19 | 45231295 | 3.2402 | Lbx1 (+4515), Tlx1 (+82060) |
| chr19  www.aging-us.com 6 AGING | 45231361 | 1.8592 | Lbx1 (+4449), Tlx1 (+82126) |
| chr19 | 45248973 | 0.0933 | Lbx1 (-13163) |
| chr19 | 45741565 | 2.1071 | Fbxw4 (-81254), Fgf8 (+1307) |
| chr19 | 46033305 | -1.7507 | Ldb1 (+11904), Hps6 (+29828) |
| chr19 | 46033385 | 3.3502 | Ldb1 (+11824), Hps6 (+29908) |
| chr19 | 46502448 | -20.3462 | Trim8 (+801) |
| chr19 | 46503408 | -2.6971 | Trim8 (+1761), Arl3 (+69676) |
| chr19 | 47068412 | 3.3484 | Taf5 (+665) |
| chr19 | 57453120 | 1.9197 | Trub1 (+215) |
| chr19 | 57611655 | -6.0035 | Atrnl1 (+622) |

www.aging-us.com 7 AGING
