# Supplementary material for: A multi-tissue full lifespan epigenetic clock for mice
Source: Aging (Albany NY). 2018 Oct 21;10(10):2832–54. doi: 10.18632/aging.101590 (PMC6224226; doi:10.18632/aging.101590)
Supplement: Supplementary Table 3 [file aging-10-101590-s004.docx]

**Supplementary Table 3.** List of strains from the Hybrid Mouse Diversity Panel (HMDP) used in the Genome-Wide Association Study.

| **No.** | **HMDP strain** |
| --- | --- |
| 1 | 129X1/SvJ |
| 2 | A/J |
| 3 | AKR/J |
| 4 | AXB1/PgnJ |
| 5 | AXB10/PgnJ |
| 6 | AXB12/PgnJ |
| 7 | AXB13/PgnJ |
| 8 | AXB15/PgnJ |
| 9 | AXB19/PgnJ |
| 10 | AXB19a/PgnJ |
| 11 | AXB19b/PgnJ |
| 12 | AXB2/PgnJ |
| 13 | AXB23/PgnJ |
| 14 | AXB24/PgnJ |
| 15 | AXB4/PgnJ |
| 16 | AXB5/PgnJ |
| 17 | AXB6/PgnJ |
| 18 | AXB8/PgnJ |
| 19 | BALB/cJ |
| 20 | BUB/BnJ |
| 21 | BXA1/PgnJ |
| 22 | BXA11/PgnJ |
| 23 | BXA12/PgnJ |
| 24 | BXA13/PgnJ |
| 25 | BXA14/PgnJ |
| 26 | BXA16/PgnJ |
| 27 | BXA2/PgnJ |
| 28 | BXA24/PgnJ |
| 29 | BXA25/PgnJ |
| 30 | BXA26/PgnJ |
| 31 | BXA4/PgnJ |
| 32 | BXA7/PgnJ |
| 33 | BXA8/PgnJ |
| 34 | BXD1/TyJ |
| 35 | BXD11/TyJ |
| 36 | BXD12/TyJ |
| 37 | BXD13/TyJ |
| 38 | BXD14/TyJ |
| 39 | BXD15/TyJ |
| 40  www.aging-us.com 1 AGING | BXD19/TyJ  www.aging-us.com 1 AGING |
| 41 | BXD2/TyJ |
| 42 | BXD20/TyJ |
| 43 | BXD21/TyJ |
| 44 | BXD22/TyJ |
| 45 | BXD24/TyJ-Cep290<rd16>/J |
| 46 | BXD27/TyJ |
| 47 | BXD28/TyJ |
| 48 | BXD32/TyJ |
| 49 | BXD33/TyJ |
| 50 | BXD34/TyJ |
| 51 | BXD36/TyJ |
| 52 | BXD38/TyJ |
| 53 | BXD39/TyJ |
| 54 | BXD40/TyJ |
| 55 | BXD42/TyJ |
| 56 | BXD5/TyJ |
| 57 | BXD6/TyJ |
| 58 | BXD8/TyJ |
| 59 | BXD9/TyJ |
| 60 | BXH10/TyJ |
| 61 | BXH14/TyJ |
| 62 | BXH19/TyJ |
| 63 | BXH2/TyJ |
| 64 | BXH20/KccJ |
| 65 | BXH22/KccJ |
| 66 | BXH4/TyJ |
| 67 | BXH6/TyJ |
| 68 | BXH7/TyJ |
| 69 | BXH8/TyJ |
| 70 | BXH9/TyJ |
| 71 | C3H/HeJ |
| 72 | C57L/J |
| 73 | C58/J |
| 74 | CBA/J |
| 75 | CE/J |
| 76 | DBA/2J |
| 77 | KK/HlJ |
| 78 | LG/J |
| 79 | LP/J |
| 80 | NOD/ShiLtJ |
| 81 | NON/ShiLtJ |
| 82 | NZW/LacJ |
| 83  www.aging-us.com 2 AGING | PL/J |
| 84 | RIIIS/J |
| 85 | SEA/GnJ |
| 86 | SJL/J |
| 87 | SM/J |
| 88 | SWR/J |

www.aging-us.com 3 AGING
